# Impacts of Antarctic summer sea-ice extremes

**DOI:** 10.1093/pnasnexus/pgaf164

**Published:** 2025-07-01

**Authors:** Edward W Doddridge, William R Hobbs, Matthis Auger, Philip W Boyd, Sean M T Chua, Sue Cook, Stuart Corney, Louise Emmerson, Alexander D Fraser, Petra Heil, Nat Kelly, Delphine Lannuzel, Xueke Li, Guillaume Liniger, Robert A Massom, Amelie Meyer, Phillip Reid, Colin Southwell, Paul Spence, Anton Steketee, Kerrie M Swadling, Nathan Teder, Barbara Wienecke, Pat Wongpan, Kaihe Yamazaki

**Affiliations:** Australian Antarctic Program Partnership, Institute for Marine and Antarctic Studies, University of Tasmania, nipaluna/Hobart, Tasmania 7001, Australia; Australian Antarctic Program Partnership, Institute for Marine and Antarctic Studies, University of Tasmania, nipaluna/Hobart, Tasmania 7001, Australia; ARC Centre of Excellence for Climate Extremes, University of Tasmania, nipaluna/Hobart, Tasmania 7001, Australia; Institute for Marine and Antarctic Studies, University of Tasmania, nipaluna/Hobart, Tasmania 7001, Australia; The Australian Centre for Excellence in Antarctic Science, University of Tasmania, nipaluna/Hobart, Tasmania 7001, Australia; Australian Antarctic Program Partnership, Institute for Marine and Antarctic Studies, University of Tasmania, nipaluna/Hobart, Tasmania 7001, Australia; Australian Antarctic Program Partnership, Institute for Marine and Antarctic Studies, University of Tasmania, nipaluna/Hobart, Tasmania 7001, Australia; Australian Antarctic Division, Kingston, Tasmania 7050, Australia; Australian Antarctic Program Partnership, Institute for Marine and Antarctic Studies, University of Tasmania, nipaluna/Hobart, Tasmania 7001, Australia; Australian Antarctic Program Partnership, Institute for Marine and Antarctic Studies, University of Tasmania, nipaluna/Hobart, Tasmania 7001, Australia; Institute for Marine and Antarctic Studies, University of Tasmania, nipaluna/Hobart, Tasmania 7001, Australia; Australian Antarctic Division, Kingston, Tasmania 7050, Australia; Australian Antarctic Program Partnership, Institute for Marine and Antarctic Studies, University of Tasmania, nipaluna/Hobart, Tasmania 7001, Australia; Institute for Marine and Antarctic Studies, University of Tasmania, nipaluna/Hobart, Tasmania 7001, Australia; Australian Antarctic Program Partnership, Institute for Marine and Antarctic Studies, University of Tasmania, nipaluna/Hobart, Tasmania 7001, Australia; Australian Antarctic Division, Kingston, Tasmania 7050, Australia; Australian Antarctic Division, Kingston, Tasmania 7050, Australia; Australian Antarctic Program Partnership, Institute for Marine and Antarctic Studies, University of Tasmania, nipaluna/Hobart, Tasmania 7001, Australia; Institute for Marine and Antarctic Studies, University of Tasmania, nipaluna/Hobart, Tasmania 7001, Australia; The Australian Centre for Excellence in Antarctic Science, University of Tasmania, nipaluna/Hobart, Tasmania 7001, Australia; Institute at Brown for Environment and Society, Brown University, Providence, RI 02912, USA; Currently at Department of Earth & Environmental Science, University of Pennsylvania, Philadelphia, PA 19104, USA; ARC Centre of Excellence for Climate Extremes, University of Tasmania, nipaluna/Hobart, Tasmania 7001, Australia; Monterey Bay Aquarium Research Institute, Moss Landing, CA 95039, USA; Australian Antarctic Program Partnership, Institute for Marine and Antarctic Studies, University of Tasmania, nipaluna/Hobart, Tasmania 7001, Australia; The Australian Centre for Excellence in Antarctic Science, University of Tasmania, nipaluna/Hobart, Tasmania 7001, Australia; Australian Antarctic Division, Kingston, Tasmania 7050, Australia; ARC Centre of Excellence for Climate Extremes, University of Tasmania, nipaluna/Hobart, Tasmania 7001, Australia; Institute for Marine and Antarctic Studies, University of Tasmania, nipaluna/Hobart, Tasmania 7001, Australia; Australian Antarctic Program Partnership, Institute for Marine and Antarctic Studies, University of Tasmania, nipaluna/Hobart, Tasmania 7001, Australia; Australian Bureau of Meteorology, nipaluna/Hobart, Tasmania 7001, Australia; Australian Antarctic Division, Kingston, Tasmania 7050, Australia; Australian Antarctic Program Partnership, Institute for Marine and Antarctic Studies, University of Tasmania, nipaluna/Hobart, Tasmania 7001, Australia; Institute for Marine and Antarctic Studies, University of Tasmania, nipaluna/Hobart, Tasmania 7001, Australia; The Australian Centre for Excellence in Antarctic Science, University of Tasmania, nipaluna/Hobart, Tasmania 7001, Australia; The Australian Centre for Excellence for Weather of the 21st Century, Institute for Marine and Antarctic Studies, University of Tasmania, nipaluna/Hobart, Tasmania 7001, Australia; Australian Antarctic Program Partnership, Institute for Marine and Antarctic Studies, University of Tasmania, nipaluna/Hobart, Tasmania 7001, Australia; Australian Antarctic Division, Kingston, Tasmania 7050, Australia; Currently at Australian Earth System Simulator (ACCESS-NRI), Australian National University, Canberra, ACT 2601, Australia; Australian Antarctic Program Partnership, Institute for Marine and Antarctic Studies, University of Tasmania, nipaluna/Hobart, Tasmania 7001, Australia; Institute for Marine and Antarctic Studies, University of Tasmania, nipaluna/Hobart, Tasmania 7001, Australia; School of Computer and Mathematical Sciences, University of Adelaide, Adelaide, South Australia 5005, Australia; Australian Antarctic Division, Kingston, Tasmania 7050, Australia; Australian Antarctic Program Partnership, Institute for Marine and Antarctic Studies, University of Tasmania, nipaluna/Hobart, Tasmania 7001, Australia; Institute for Marine and Antarctic Studies, University of Tasmania, nipaluna/Hobart, Tasmania 7001, Australia; The Australian Centre for Excellence in Antarctic Science, University of Tasmania, nipaluna/Hobart, Tasmania 7001, Australia

**Keywords:** sea ice, Southern Ocean, Antarctica, extremes, impacts

## Abstract

Antarctic sea ice plays many crucial roles in the physical environments and ecosystems of Antarctica and the Southern Ocean. In this study, we synthesize the physical, biogeochemical, ecosystem, and societal impacts of summers with extreme low Antarctic sea-ice coverage. These extreme events result in the loss of multiyear landfast ice and changes in sea-ice seasonality. Following extreme low sea-ice events, we find surface warming of the Southern Ocean and changes to the formation rate of Antarctic Intermediate Water, likely affecting heat and carbon uptake. Ice-shelf calving is negatively correlated with sea-ice area, so that years with less sea ice show increased calving. Prolonged open water affects the magnitude and seasonality of surface-phytoplankton blooms. The impacts on higher trophic levels are species-specific and occur through habitat loss and changes to prey availability. Extreme sea-ice lows will adversely impact krill, a foundational prey species that relies on sea ice for nourishment and refuge. The loss of stable landfast ice in austral spring and summer hampers Antarctic operations and resupply missions. Understanding the full impacts of recent, and future, sea-ice extremes is of utmost importance and requires an enhanced observational network that spans the physical and ecological systems of Antarctica and the Southern Ocean.

Significance Statement Antarctic sea ice appears to be changing; in the last decade, we have observed both record highs and record lows in Antarctic sea ice coverage. This article addresses the impacts of extreme lows in Antarctic summer sea ice coverage. We find that the impact of an extreme event is larger than one would expect from linear extrapolation. Furthermore, we find that these extreme events have substantial impacts across physical, ecological, and societal systems, from ocean warming and increased iceberg calving rates, through to habitat loss for higher-order predators, and logistical challenges for national Antarctic programs. Understanding, and predicting, the impacts of these extreme events requires long-term monitoring.

## Introduction

Sea ice forms at the interface between atmosphere and ocean. It is a seasonally variable, and crucial, part of the climate system, playing many key roles in the Southern Ocean and its ecosystems. Recent decades have seen a dramatic positive then negative shift in Antarctic sea-ice area—with successive record winter highs in 2012, 2013, and 2014, followed by three record low summers in 2017, 2022, and 2023 (1 ). This variability is unprecedented in an observational record dating back to 1978 (2, 3). In addition to this increase in variability, Antarctic sea ice entered a new low-extent regime in September 2016 (4) and recent extreme lows have exhibited a decrease in spatial heterogeneity, with reductions in sea-ice extent occurring consistently around the continent (5). Recent summer events provide a window into the likely future in a warmer climate with reduced average sea ice (6–8), and increased likelihood of extreme events in various systems (9 ). The combination of a projected decrease in the mean state of sea ice and the observed increase in variability raises the prospect of low sea-ice events becoming increasingly extreme and frequent.

Antarctic sea ice provides climate and ecosystem services of regional and global significance. The high albedo of its near-ubiquitous snow cover makes sea ice important in setting local ocean mixed-layer temperature, and the global energy balance (10, 11). This is augmented by the freeze/melt cycle’s role for Southern Ocean uptake of heat and climate active gases, including CO_2_ (12, 13). Sea ice protects Antarctic ice shelves from potentially damaging ocean swells (14 ), which has implications for global sea-level rise, and provides habitat for a range of ice-dependent organisms and ecosystems on and around Antarctica (15).

Because the coupled climate system contains numerous feedback mechanisms and nonlinear interactions, the impact of an extreme change in sea-ice coverage may be significantly larger than linear extrapolation would suggest. Within the physical climate system, sea ice reflects much more incoming solar energy than open water, such that sea-ice loss can lead to ocean warming via the ice-albedo feedback mechanism, accelerating sea-ice loss (16). The interactions between sea ice and biological systems are highly complex, but observations of extreme sea-ice loss leading to catastrophic breeding failures highlight the risk that entire ecosystems may struggle to adapt in the face of rapid change and increased variability (17, 18).

The ocean provides a memory to the coupled ocean—sea ice—atmosphere system, meaning that sea-ice and ocean-temperature anomalies may survive for several years (19). If multiple sea-ice extremes occur within a persistence window, then there is potential for their impacts to compound. In the physical system, this may manifest as persistent changes in ocean temperature or salinity, while the ecological response may manifest as repeated years with high mortality or low breeding success, potentially culminating in large demographic shifts.

Our knowledge of the southern polar region, and the ecosystems it supports, is increasing. However, we do not yet have sufficient understanding of the baseline system to be able to predict how it will respond to the dramatic changes we are already observing. In order to predict future changes, and to potentially mitigate the negative impacts of climate change on this region, we urgently need to improve our knowledge through additional observations and modeling studies of the physical and biological systems around Antarctica.

We seek to understand the physical, biogeochemical, ecological, and societal impacts of recent sea-ice extremes on the natural systems in and around the Southern Ocean. In this article, we combine observations, reanalysis data, and modeling to give an overview of observed and potential impacts of Antarctic sea-ice extremes, highlighting critical knowledge and data gaps that should be a priority for the polar scientific community.

## Observed changes and associated impacts

### Observed changes

**Fig. 1.**
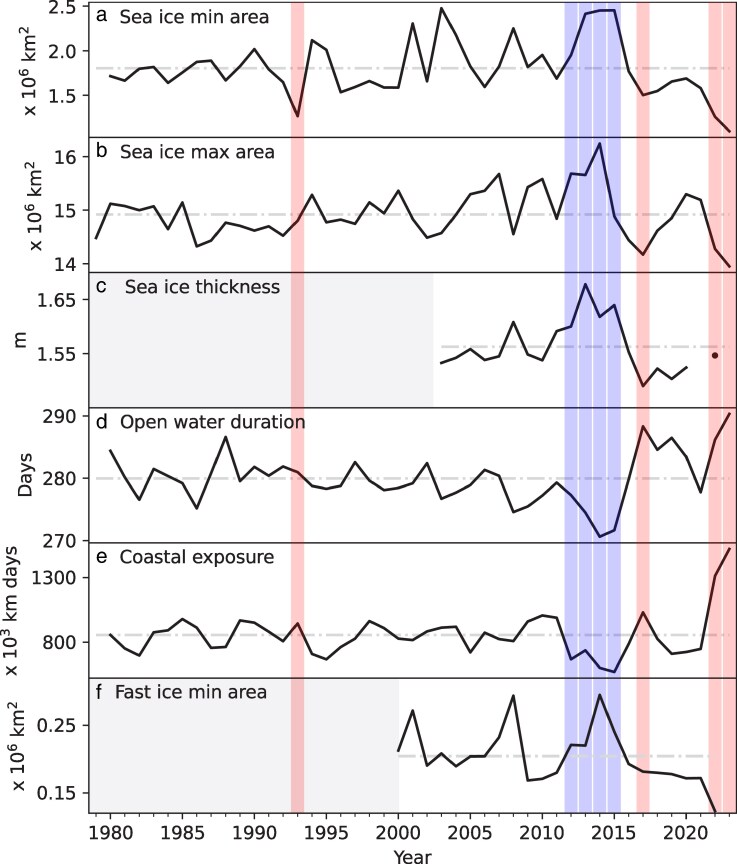
Satellite-derived net circum-Antarctic time series of annual sea-ice parameters for the period 1979–2023. a) Minimum sea-ice area, b) maximum sea-ice area, c) mean sea-ice thickness (May–October), d) mean open water duration, e) annually accumulated coastal exposure length between sea-ice maxima, and f) minimum landfast ice area. Blue and red bands identify high and low sea-ice extreme years, respectively, according to the ranking procedure given in the Supplementary Information. Grey blocks indicate data not available for those time periods. d and e) Summertime quantities, which are labeled using the convention where “2016” refers to the 2015/2016 summer, are represented.

We begin by exploring the relationship between extremes in sea-ice area and other physical sea-ice parameters (Fig. 1). In our broad-scale analysis, we consider only circum-Antarctic quantities. This is motivated by our focus on large-scale impacts, rather than regional effects, and by the reduction in sea-ice spatial variability observed in recent years (5). We also highlight the incomplete characterization of key physical sea-ice properties in Fig. 1. Knowledge of large-scale snow thickness, ice deformation, roughness, and the extent of the marginal ice zone are all required for more complete physical characterization, but long-term datasets of these metrics are lacking.

Figure 1 shows annual time series of six key sea-ice variables. Since 2008, there has been an apparent shift toward both extreme high and low sea-ice years (3, 4, 20). Figure 1 highlights the unprecedented low sea-ice coverage in 2022 and 2023 (21). These extreme events are consistent across multiple sea ice metrics, indicating the pervasive nature of these conditions across the Antarctic climate system.

Changes to the sea-ice system also manifest as changes in the duration of time where sea ice is not present in the Southern Ocean (22, 23). The recent period of low sea-ice area (2016–2022) occurs in tandem with longer open water duration, whilst the earlier period of high sea-ice area (2007–2015) occurs in tandem with shorter open water duration (Fig. 1d), consistent with previous studies of long-term trends in sea-ice advance and retreat (22, 24).

Change in sea-ice area (Fig. 1a and b) has major implications for exposure of Antarctica’s coastal margins to open-ocean conditions (Fig. 1e). Over the period 1979–2020, about 35% of the coast is exposed on average at its annual maximum each February (25). In comparison, during 2022 and 2023, approximately 53% of the coast was exposed at the February maximum. We present coastal exposure length-days, i.e. the time-integral of coastal exposure length between subsequent sea-ice maxima. Similar to other metrics, during high sea-ice years, there is less coastal margin exposed, whilst during low sea-ice years, there is more coastal margin exposed. Recent years of high coastal exposure have increased exposure of ice shelves to ocean swell, as discussed in Impacts on ice shelves section.

We also consider the total landfast sea-ice area at the time of minimum extent (Fig. 1f), which occurs in early-mid March in most years (26). Landfast ice forms in many coastal regions on the Antarctic continental shelf. It protects ice shelves from wave damage (see Impacts on ice shelves section), is a key habitat for Antarctic species (see Impacts on marine predators and their prey section), and is used as a logistics platform (see Socio-economic and well-being impacts section). Yearly minimum landfast ice extent declined to unprecedented lows in 2022 in almost all regions (27), after showing resilience for the period 2000–2018 (26). The recent decline in landfast ice area mainly stems from a loss of old (>3 years) landfast ice (see Fig. S1). Based on a dataset spanning 2000–2022 (27), much of this ice broke out for the first time in 2022 thereby exposing previously protected parts of the Antarctic coast to the ocean.

May-October mean circum-Antarctic thickness (28) is shown in Fig. 1c. Whilst this is a wintertime quantity and subject to considerable uncertainty, particularly in regions of complex snow stratigraphy (29), reliable summertime Southern Ocean sea-ice thickness estimates have yet to be produced from satellite altimetry. Our analysis shows that thinner ice occurs during recent extreme low sea-ice years (Fig. 1c), suggesting sea-ice volume loss in these years, as opposed to a dynamic thickness change compensating for a loss of sea-ice area. However, a more robust investigation requires a longer time series, along with Antarctic-specific in situ validation of satellite derived thickness measurements.

### Oceanographic impacts

Sea ice has a strong control on near-surface Southern Ocean temperature. In summer the primary heat source is the sun, and the high albedo of snow-covered ice means that sea ice coverage is the dominant constraint on summer ocean temperature (30). In winter, the primary heat source is entrainment of relatively warm deep water into the mixed layer through brine rejection during sea ice formation (31). Winter entrainment also erodes thermal memory in the ocean mixed layer, and means that under normal circumstances sea ice anomalies do not persist from one summer to the next (19).

Recent extremes in sea ice have occurred alongside a number of changes to the Southern Ocean. Observations from the global Argo array show that the subsurface Southern Ocean has warmed and become saltier (4, 32). Following recent extremes, there is warming and salinification to at least 400 m. This anomaly is surface-intensified, strongly suggesting a response to sea ice cover changes. In particular, the extreme warming observed in the 2016/2017 summer coincides with a record breaking summer minimum with sea ice extent dropping to its then lowest value since the beginning of the satellite record. This record has subsequently been broken in 2022 and again in 2023, while the subsurface ocean continues to warm.

Ocean observations show a near-surface Southern Ocean warm anomaly since the sudden sea-ice decline in 2016 (4). However, it is impossible to fully assess the high latitude impacts of summer sea-ice loss from observations alone: there are very few ocean profiles on the continental shelf and those that exist are biased towards the summer period, precluding an assessment of impacts sustained across seasons; and observed changes will include the imprint of all physical drivers (e.g. atmospheric variability) not just the effects of sea ice. Therefore, to estimate the ocean response to extreme lows in sea ice, we turn to numerical experiments.

**Fig. 2.**
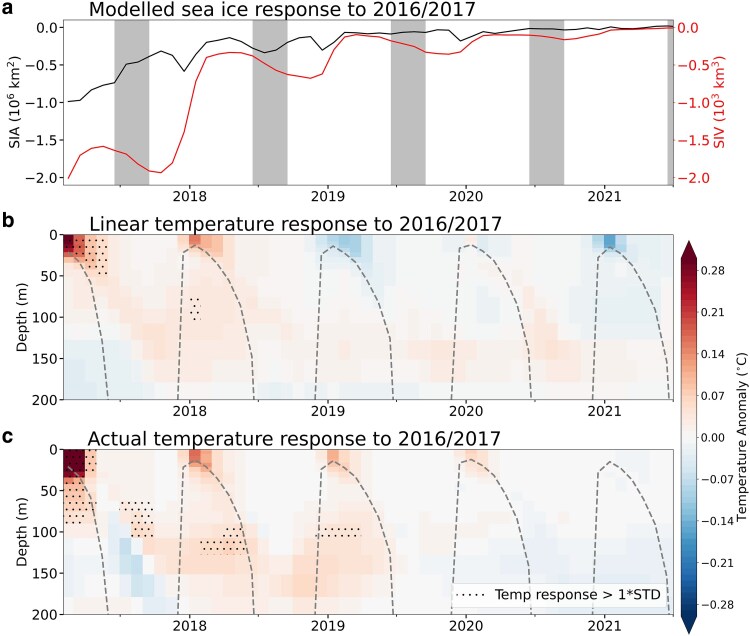
Sea ice and ocean temperature response to the 2016/2017 extreme low in summer sea ice from the ACCESS-OM2-025 model. a) Antarctic sea ice area (SIA: black) and volume (SIV: red) responses to extreme sea ice loss leading up to the record low in summer 2016/2017 estimated as the Control minus the No_2016 simulation. b) Expected temperature response to summer 2016/2017 sea ice conditions estimated from a lagged regression analysis of summer sea ice area and ocean temperature. The regression coefficients were computed based on temperature and sea ice area from 1980 to 2014. c) Temperature anomalies from the beginning of 2017 onwards. Anomalies are the Control minus the No_2016 temperature, showing the long term impact of extreme sea ice loss on the subsurface temperature field. Hatching signifies anomalies exceed one standard deviation from the 1980–2014 mean. The gray bars in a) indicate winter. Ocean plots are area-weighted circumpolar averages 65–80° S, and the dotted line in b) and c) represents the areal-mean mixed layer depth.

Analyzing the control run of our simulation shows that circumpolar ocean temperature and salinity anomalies associated with summer sea ice anomalies generally last only 12 months (Fig. 2b). However, the circumpolar oceanic response to the 2017 extreme low sea ice event lingers for three years (Fig. 2c). This suggests that the oceanic response to sea ice extremes is nonlinear; the impact of an extreme low is not just an amplified version of its response to a moderate low. Interestingly, the simulations show that this persistence is not represented in the sea ice area but is very evident in sea ice volume (Fig. 2a), implying that the observational record of sea ice area (Fig. 1) may under-represent the true impact of extreme low summer events. The volume anomalies peak in winter and are out of phase with the summer-peaking ocean temperature anomalies. In winter, the mixed layer temperature must approximate the freezing temperature but thermal anomalies are expressed as a reduction in sea ice volume; in spring this reduced volume melts faster, leading to warm surface anomalies the next summer. Following an extreme sea ice low, heat anomalies penetrate below the areal-mean summer mixed layer depth (Fig. 2c). These deeper anomalies are then entrained back into the mixed layer by brine rejection during winter ice production (19) and may contribute to the maintenance of a warmer mixed layer-sea ice system.

The seasonal cycle of sea ice plays a central role in the overturning circulation of the Southern Ocean by modifying the density of surface waters (12) (Fig. S2). Our numerical simulations show that extreme sea ice loss results in a reduction in the conversion of Circumpolar Deep Water into Antarctic Intermediate Water (Fig. S2), and thus a reduction in the ventilation rate of the ocean interior north of the Antarctic Circumpolar Current.

### Impacts on ice shelves

Extreme low sea ice events lead to warmer Antarctic surface waters (Oceanographic impacts section) and increased exposure of the Antarctic coastline to ocean swell (Observed changes section). Both of these impacts have the potential to affect mass loss from Antarctic ice shelves: changes in ocean temperature affect the rate of ocean-driven ice-shelf melt (33), and exposure to ocean swell is hypothesized to affect iceberg calving rates (14). Processes affecting mass loss from ice shelves are important for understanding the future contribution of the Antarctic Ice Sheet to global sea level, because the ice shelves provide a “buttressing” force which slows the flow of grounded ice from the interior of the continent into the ocean (34).

Warm summer surface water can be drawn under the ice-shelf front, driving increased melt at shallow depths. This process has been observed on the Ross Ice Shelf, where basal melt rates almost tripled during summer months, although the effect was restricted to within a few kilometers of the ice shelf front (35). In front of the Amery Ice Shelf, anomalously warm summer surface water associated with an anomalously low sea ice extent in summer 2016/2017 caused enhanced ice shelf melting at shallow depths and delayed the onset of freezing in the neighboring polynya by 3 weeks (36).

Results from the ACCESS-OM2-025 perturbation experiment (Oceanographic impacts section) show that the extreme low sea ice area in 2016 caused significant warming of coastal surface waters (Fig. S3). The summer warming anomaly was found to persist for several years, although the very intense ocean warming is confined to the shallowest 150 m (Fig. 2), which is likely to limit its impact on the wider ice shelf cavity, as typical ice shelf drafts are over 400 m. Previous studies have found that melt driven by surface waters is not currently a dominant mode of melt in Antarctic ice shelves (37, 38). However, these modeling studies also indicate that this mode of melting could become more important in a future with reduced summer sea ice cover.

Sea ice loss can also increase melt rates through ocean dynamics. Recent observations of the Fimbul ice shelf show that with less ice cover, winds have more effect on the local ocean circulation and can change the rate of warm water transport to an ice shelf (39). Changes in sea ice production can also impact basal melt rates, since the Dense Shelf Water formed by sea ice production in coastal polynyas provides a measure of protection from warm Circumpolar Deep Water for some ice shelves (38, 40). The ACCESS-OM2-025 perturbation experiment showed no change in dense water formation near the coast (Fig. S2). However, the ACCESS-OM2 model does not include ice shelves and is too coarse to represent the small spatial scales of polynya-ice shelf interaction. The impact of changes in sea ice on deeper water masses will vary both spatially and temporally, and requires high-resolution ocean models with ice shelf cavities to be studied in detail.

Sea ice acts as a protective barrier to an ice shelf by attenuating incoming ocean swell (41). A significant loss of sea ice allows ocean swell to induce flexural strains in the outer margins of the shelf (14), which contributes to the expansion of nearby fractures and a general fatiguing of the ice shelf (42). The loss of regional sea ice cover has been linked to ice-shelf disintegrations on the Antarctic Peninsula (14) and has been hypothesized to be a trigger mechanism for iceberg calving (43). Recent work has quantified the exposure of ice shelves to ocean swell by detecting sea ice-free corridors based on sea ice concentration data, and using them to measure the number of days per year ice shelves around Antarctica are exposed to large ocean swell (44).

**Fig. 3.**
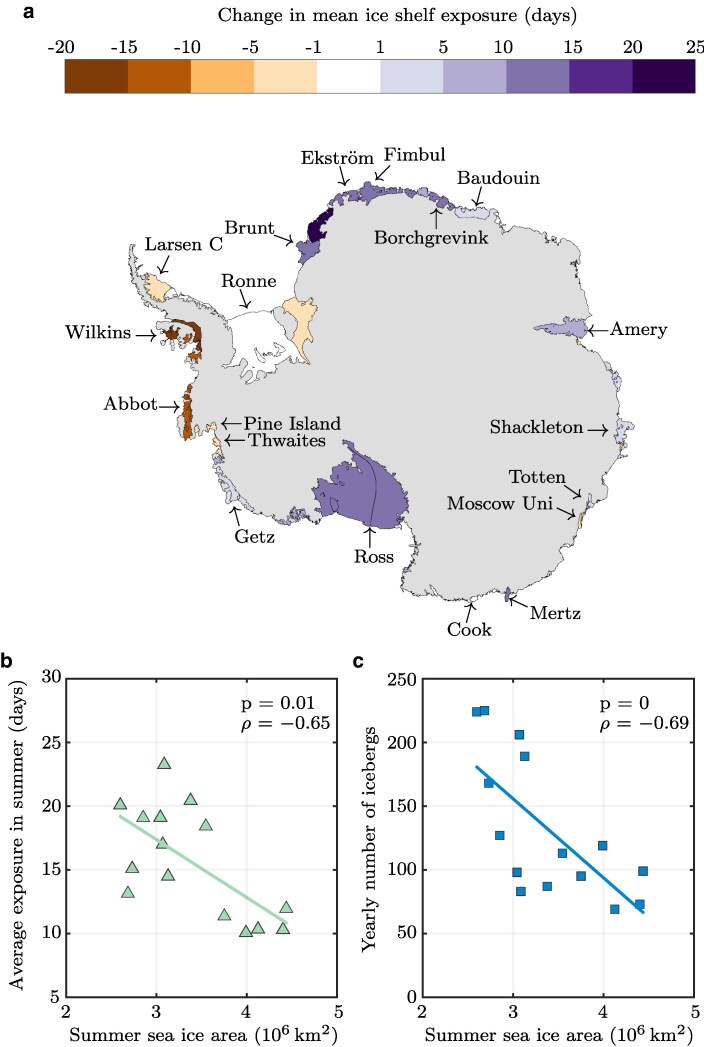
Relationship between summer-averaged sea ice area, ice shelf exposure, and yearly iceberg calving. a) Ice shelf exposure days averaged over the five lowest sea ice area years, compared to the long-term mean (2006–2019). b) Summer sea ice area vs days of ice shelf exposure. c) Summer sea ice area vs number of iceberg calving events per year.

We find a significant negative correlation between circumpolar summer sea ice area and ice-shelf exposure, confirming increased exposure of ice shelves to ocean swell in low sea ice years (Fig. 3a and b, Table S1). The strongest effect is found between Brunt and Borchgrevink ice shelves, as well as at the Mertz and Ross ice shelves where low sea ice area anomalies resulted in an increase of >10 days per year in ice-shelf exposure, with sea ice-free corridors forming earlier in the year (44). Areas of low correlation may indicate regions of locally heterogeneous sea ice behavior. For example, a break up in the Wilkins Ice Shelf in 2008/2009 coincided with anomalously low sea ice concentrations locally, which were not reflected in total sea ice area (14).

We also find a statistically significant relationship between continent-wide summer sea ice area and number of iceberg calving events, confirming that low sea ice years do result in increased iceberg calving frequency as hypothesized (Fig. 3c, Table S1). Our results show that a decrease of 100,000 km^2^ in summer sea ice area leads to approximately six additional icebergs that year. Due to the short observational record, as well as the wide range of factors that can influence iceberg calving (e.g. surface and basal melt, (45)), the statistical relationship here is weaker. Nonetheless, these results support the previously hypothesized mechanism linking sea ice-free corridors and iceberg calving. This effect may also be enhanced by the recent decrease in landfast sea ice (Figs. 1f and S1) that occurred during record high coastal exposure in 2022 (27). While the available records of iceberg calving do not yet cover 2022, break-up of landfast sea ice has previously been linked to both increased ice shelf velocity and calving events (46–48) and is anticipated to exacerbate ice shelf mass loss.

### Impacts on primary productivity

The marginal ice zone is the wind- and wave-fractured region of unconsolidated sea ice floes between the sea ice edge and the interior pack. The location and size of the marginal ice zone varies during both growth (advance) and melt (retreat) seasons. It is a key biogeochemical and biological region of the Southern Ocean where ice-edge blooms typically occur within 1 to 2 weeks of sea-ice retreat. Marginal ice zone phytoplankton blooms are likely driven by a combination of enhanced stratification and the delivery of dissolved iron and algae by sea ice melt (49). In this context, episodes of earlier and faster sea-ice retreat can lead to increases in the areal extent of co-located buoyancy and dissolved iron supply, potentially changing the timing and magnitude (phenology) of algal (phytoplankton and ice algae) blooms in the Southern Ocean.

Modeling suggests that marginal ice zone blooms account for 15% of yearly net primary production in the Southern Ocean (50) and that the early phases of the marginal ice zone blooms, which occur under sea ice and are therefore invisible to satellite remote sensing, account for about two-thirds of this production. This highlights the need for a combination of both satellite and water column views of the marginal ice zone system. In polar regions, episodes of under-ice biological productivity have been reported (51–53). Notably, BioGeoChemical Argo floats (BGC-Argo) have proven to be valuable tools to detect under-ice biogeochemical processes in polar regions (54–57). Very recently, these platforms reported events of high phytoplankton biomass (with maximum chlorophyll-*a* concentrations varying from 0.1 to 3.5 mg/m^3^) below Antarctic sea ice (58–61) suggesting that more than 4 million km^2^ of Antarctic sea ice cover could support under-ice blooms in late spring and early summer. Under-ice blooms may in turn influence the carbon cycle through enhanced downward export fluxes (62), and the lower atmosphere via the production of new particles for nucleation and marine cloud formation (through release of volatile organic compounds; see Refs. (63, 64)).

**Fig. 4.**
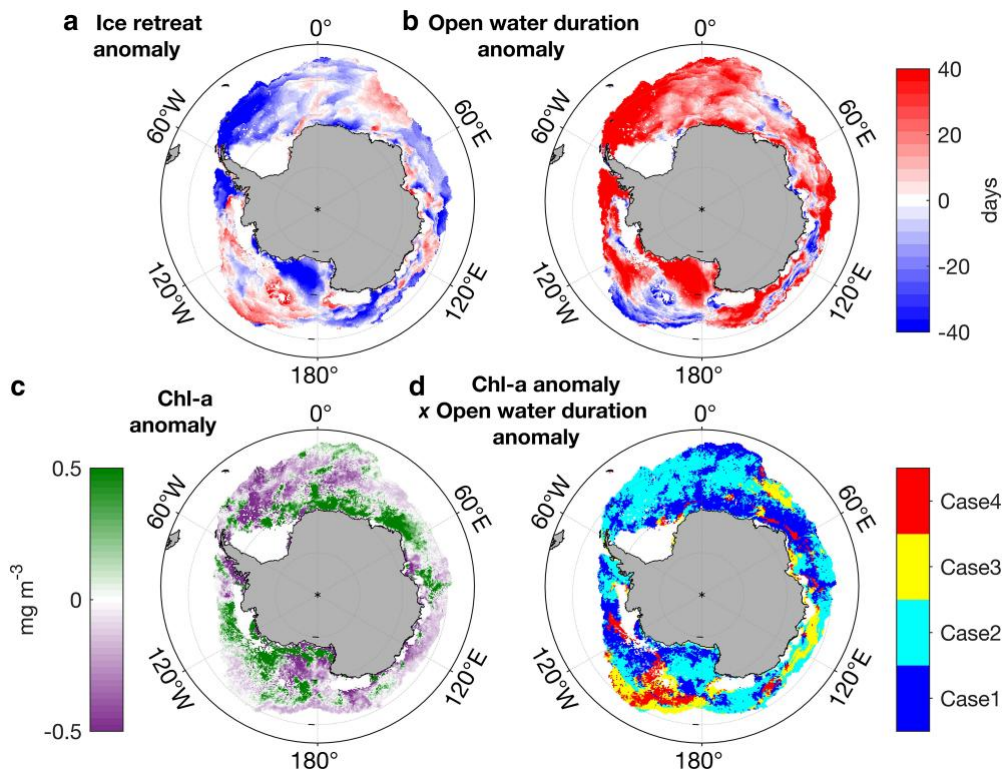
2021/2022 anomalies in a) ice retreat time (negative means earlier), b) open water duration (OWD), c) surface chlorophyll-*a*, and d) relationship between anomalous surface chlorophyll-*a* and OWD. Case 1 = longer OWD and higher chlorophyll-*a*; Case 2 = longer OWD and lower chlorophyll-*a*; Case 3 = shorter OWD and lower chlorophyll-*a* and; Case 4 = shorter OWD and higher chlorophyll-*a*. 2021/2022 anomaly was compared against the 2004/2018 time period. The light black ticks represent the 10° latitude mark.

Satellite observations during the 2021/2022 summer revealed contrasting responses from phytoplankton to changes in sea-ice retreat time and open water duration (Fig. 4). Areas of earlier sea ice retreat (Fig. 4a) and longer open water duration (Fig. 4b) translated to lower chlorophyll-*a* (Fig. 4c) offshore in the Ross and Weddell seas, but higher chlorophyll-*a* in King Haakon VII and coastal Ross Sea zones (Fig. 4d). For the 2021/2022 event, we used the only available BGC-Argo float profiles (float #7900673) located at the time in the marginal ice zone of the Ross Sea to evaluate whether differences in the mixed layer depths matched the contrasts in satellite ocean color (chlorophyll-*a*) between regions, as chlorophyll-*a* was not available for that float. The BGC-Argo float revealed a freshening signature between the 2020/2021 and 2021/2022 sea-ice seasons (Fig. S4, stronger signal in 2021/2022 season). Float profile #220 showed the greatest freshening (Fig. S4), a signal that was co-located with an anomalously high satellite chlorophyll-*a* retrieval (Fig. S5) and anomalously longer open water duration. Satellite chlorophyll-*a* estimates matching the location of the late February profile (#220) during the 2021/22 season is 1.39 mg m^−3^, representing an anomalous increase of 0.39 mg m^−3^ above the 2004-2018 baseline value (1 mg m^−3^). Using 0.81 μg chlorophyll-*a* synthesized per nanomole of dissolved iron (based on a polar front study (65)), and estimates of dissolved iron supplied from sea ice meltwater (Table S2), the freshening observed from the BGC-Argo float could translate into a chlorophyll-*a* increase of 0.34 ± 0.53 mg m^−3^ for profile #220 (Table S2). The BGC-Argo derived chlorophyll-*a* increase estimate is very close to the satellite chlorophyll-*a* estimate and falls into its uncertainties range (0.34 ± 0.53 mg m^−3^ for the BGC-Argo, compared to 0.39 mg m^−3^ for satellite). The BGC-Argo fleet in the Southern Ocean was smaller in 2016/2017 compared to 2021/2022. The few floats located in the vicinity of the marginal ice zone show contrasting results. In the Ross sea, float #5904180 highlighted a stronger bloom in 2016/2017 compared to 2015/2016, associated with fresher conditions and a shallower mixed layer (Fig. S6a, c, and e), despite slightly above average sea ice coverage (Fig. S7). On the other hand, float #5904397 in the King Haakon VII Sea also detected a longer and stronger freshening in 2016/2017 but did not translate to higher productivity compared to 2015/2016 (Fig. S6b, d, and f) while sea ice was anomalously low for that time (Fig. S7).

Our results highlight the complex interactions between ocean, cryosphere, and primary production. While both events show an increase in productivity associated with stronger freshening, contrasting regional trends are also observed. Therefore, it is not possible to ascribe this outcome to the anomalous sea-ice coverage alone.

The discrepancies observed in our study and previous studies (e.g. (66)) may be caused by natural interannual variability, light availability or nutrient replenishment by deep-mixing (67–70). For instance, the mismatch between the lower satellite chlorophyll-*a* and longer open water duration in the Weddell Sea for the 2021/2022 could be caused by a deeper mixed layer and/or lower light availability. Satellites are limited to the 1st optical depth and also generally cannot see through sea ice or clouds, limiting observations to surface ocean chlorophyll-*a* under blue skies. Overall, our results highlight the importance of continuing and improving in situ monitoring using all available tools (e.g. BGC-Argo, animal-borne sensors, moorings, and autonomous underwater vehicles) complemented with process studies to properly evaluate the Antarctic phytoplankton response to sea-ice changes on different spatial and temporal scales.

As well as impacting open ocean phytoplankton growth, changing sea-ice conditions can also affect algal primary production within sea ice (18). Sea-ice algal productivity cannot be quantified remotely (via satellites or floats) and large uncertainties remain, with sea-ice biogeochemical models and field-based observations reporting a wide range of Antarctic sea-ice primary production estimates, from 15.5 to 70 TgC y-1 (71–74). Our understanding of the importance of sea ice for primary productivity is severely hindered by the widespread under sampling of integrated physical and biogeochemical variables in the seasonal sea ice zone (75). The impact of summer sea ice extremes on total primary productivity is unclear, with the net outcome of changes in sea ice and ocean productivity unknown.

### Impacts on marine predators and their prey

The Southern Ocean supports a diverse range of iconic, higher-order predators. Species vary in their breeding and foraging behaviors, the timing of their life cycle stages, migration patterns, and habitat requirements. As such, their responses to sea ice extremes are species-specific (76, 77). Extreme sea ice events affect predators directly through changes to their sea ice environment, and indirectly through the association between sea ice and their prey (78–80). Species that breed and forage throughout their life cycle within the Antarctic sea-ice zone (e.g. emperor penguins *Aptenodytes forsteri* and crabeater seals *Lobodon carcinophagus*) have different vulnerabilities to extreme events than species that breed on ice-free land, and during winter either remain within the sea-ice zone (e.g. Adélie penguins *Pygoscelis adeliae*, snow petrels *Pagodroma nivea*), or migrate beyond it (e.g. cape petrels *Daption capense*). Several species breeding outside the Antarctic zone utilize the rich Southern Ocean resources during the summer (e.g. shearwaters *Ardenna* spp., Arctic terns *Sterna paradisaea*, and Antarctic minke whales *Balaenoptera bonaerensis*) and rely on the presence of productive open water during summer.

#### Direct physical interactions and dependencies between predators and sea ice

**Fig. 5.**
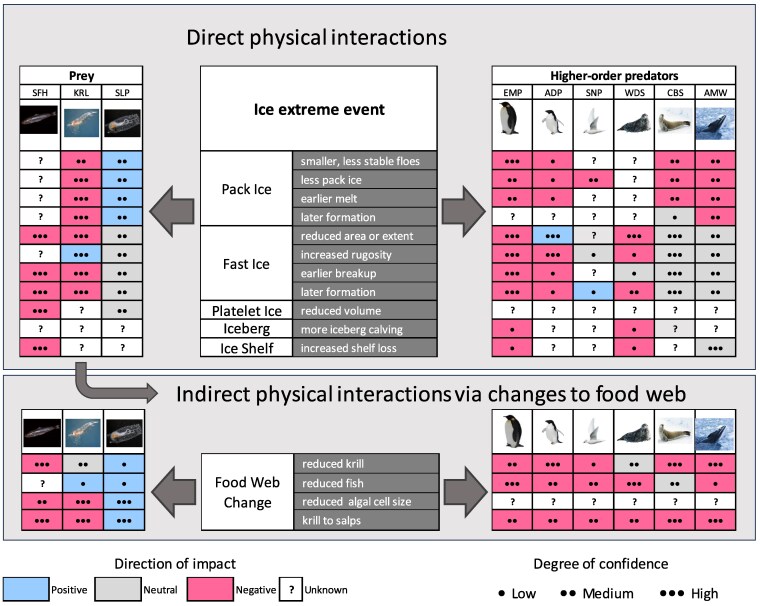
Generalized response of higher-order predators and their prey to extreme events associated with sea ice. Response scores and confidence are based on the consensus of authors’ expert knowledge of species biology and ecology, and are combined scores for impacts on survival, breeding success, population trajectory, and habitat change. Responses indicate likely outcomes relating to direct interactions between predators or prey and the sea ice environment. Responses to food web change indicate indirect interactions. These can vary in direction and magnitude, particularly in relation to limiting factors for each population. Given the sea ice conditions in East Antarctica, it is expected that the ice-breeding species are likely to respond negatively to sea ice loss. Where the ice provides a barrier to prey access, less sea ice can be of benefit. Species molting on sea ice are likely hampered by either rapid melt or loss of pack ice when they are using it as a platform. The cumulative impact of extreme sea ice events is an outcome from direct interactions with the sea ice and indirect interactions through the impact of sea ice conditions on the predators prey field. The acronyms used in the figure are: SFH, Antarctic silverfish; KRL, Krill; SLP, Salp; EMP, Emperor penguin; ADP, Adélie penguin; SNP Snow petrel; WDS, Weddell seal; CBS, Crabeater seal; AMW, Antarctic minke whale.

Higher-order predators display species-specific dependencies and interactions with sea ice that govern their responses to extreme ice events (Fig. 5).

Long-lived predators are likely to experience population reductions if extreme events cause widespread, high mortality of adults, which in turn reduces reproduction in the following years. Particularly vulnerable are species that breed or molt on sea ice. For example, like all penguins, emperors undergo a “catastrophic molt” during which the entire plumage is replaced in a few weeks. When molting, they must stay out of the water as their plumage is no longer waterproof (81). Similarly, all ice-breeding seal species (crabeater, leopard, Ross, and Weddell) haul out on ice floes to replace their fur. Although seals utilize floes of varying sizes, larger floes provide not only a suitable molt platform but also protection from marine predators, such as leopard seals *Hydrurga leptonyx* or killer whales *Orcinus orca* (82). Extreme lows in summer sea ice may reduce the availability of suitable ice floes to critically low levels potentially threatening the survival of seals and penguins molting in the pack ice zone. Likewise, decreased sea ice cover may increase the predation risk for Antarctic minke whales *Balaenoptera bonaerensis*, as they use pack ice to shelter away from their key predator, “Type A” killer whales, which are typically observed only in open water (83). In this context, flying seabirds are less vulnerable to extreme sea ice events because they molt continuously over several months during the summer and autumn.

Breeding crabeater seals avoid small, unstable ice floes in spring for the 2–3 weeks from giving birth to weaning their pup (84). At this critical time, extremely low pack-ice cover, especially in combination with increased storm and swell activity, may decrease floe sizes, break up large floes, and decrease floe stability, increasing the predation risk from leopard seals and pup mortality (82). Land-breeding flying seabirds can fly over extensive or consolidated sea ice to access foraging habitat (85) and are likely less vulnerable to extremes in landfast ice extent. However, high sea-ice concentrations over large areas may increase their foraging costs and reduce breeding success (86). When sea ice extent is reduced, species migrating from northern latitudes to forage in the Southern Ocean during the austral summer have to commute further to their foraging grounds, possibly with energetic consequences (87).

While sea ice dependent species consistently respond negatively to the loss of suitable ice floes during the molt, the effects of extreme landfast ice on reproductive success are species-specific (Fig. 5). For example, extensive landfast ice may increase travel times and trip durations between breeding and foraging habitats of Adélie and Emperor penguins (77, 88). Furthermore, the grounding of giant icebergs during chick rearing can rapidly amplify detrimental barrier effects and increase chick mortality as chicks are fed less frequently (77, 89–91). Loss of landfast ice area or integrity can cause reproductive failure among emperor penguins when the breeding platform is lost before the chicks are able to go to sea (e.g. (17, 92)). Weddell seals *Leptonychotes weddellii* also breed on the landfast ice. Since they use cracks to move between their breeding and feeding habitats, increases in the extent or thickness of landfast ice may cause the cracks to close, limiting the seals’ access (82, 93). Occasional breeding failures may reduce the size of a cohort, but these failures can often be compensated for in subsequent successful breeding seasons. However, multiple years of reproductive failure will lead to population decline (e.g. (94, 95)).

#### Impacts of extreme events on predator prey field

An important unknown for predicting wildlife responses to extreme conditions is the indirect link with prey. Prey availability is a function of the amount of prey present (prey abundance) and accessibility (79). While accessibility can relate to the physical presence of sea ice, prey abundance is likely dependent on a range of conditions, including the seasonal extent, ice concentration and degree of deformation (rafting and ridging), and the timing of formation and retreat of sea ice that may occur long distances from the breeding areas (76, 79).

Two important, high-biomass prey species in the Southern Ocean are Antarctic krill *Euphausia superba* and Antarctic silverfish *Pleuragramma antarcticum*, both closely associated with sea ice, particularly during their early development. Larval krill lack sufficient energy stores (lipids) to survive their first winter without eating (96, 97). Complex sea-ice structures provide food sources during periods of scarcity, and protection from predators (98–102). Krill recruitment (i.e. development into adulthood) depends on sea-ice conditions in the previous winter; reduced sea-ice extent compromises larval survival, and the population is reduced in the following summer (103). Near the western Antarctic Peninsula, in years when the Southern Annular Mode is positive, the flow of warm, north-westerly winds reduces winter sea-ice extent and duration, decreasing diatom biomass and krill recruitment (103). Since krill live for at least 5 years, 1 year of poor recruitment may not be detrimental. However, when adverse conditions (such as positive Southern Annular Mode) prevail for several seasons, krill will find it increasingly difficult to maintain the population. The Southwest Atlantic, which holds >50% of the circumpolar krill stock, provides a clear example of the impact of longer term, climate driven population change. Due to warming in this region (20° W–80° W), krill have contracted their main distribution southward by 440 km over the past 90 years, becoming centered more strongly over Antarctic continental shelves (104). To date, there are no established links between decreasing ice extent and krill populations for other regions of the Southern Ocean (105).

Antarctic silverfish are a lipid-rich pelagic species that comprises up to 90% of the mid-water fish biomass in the Ross Sea. They are prey for Weddell seals, whales, Adélie and emperor penguins, and flying seabirds (106). Antarctic silverfish have a circum-Antarctic distribution, yet little is known about their relationships with sea ice outside of the Ross Sea and the Antarctic Peninsula. To date, the only known Antarctic silverfish hatching ground was found in the platelet ice layer under the fast ice at Terra Nova Bay (Ross Sea) (107, 108), where embryos and larvae find protection from predators (109). Loss of habitat and the concomitant reduction in silverfish stocks may force predators to travel farther offshore to obtain access to sufficient prey that do not rely on sea ice (e.g. myctophids (lanternfish)) (110).

In landfast and pack ice areas, small invertebrates live in the brine channel system and at the under-ice surface. These invertebrates support fish that thrive in the pitted under-surface of ice, where they find refuge from predators (111). Notothens (cod icefish) are prey for emperor penguins and Weddell seals, and, while these fish can consume benthos-derived food (food found on the sea floor), their diets are primarily dependent on sea-ice associated (= sympagic) copepods whose life history strategies are tied to the cycle of growth and decay of sea ice. Qualitative modeling suggests that the reductions in sea ice duration and volume during extreme summers (Fig. 1) will negatively effect these animals (112) as they will be unable to complete their life cycles. Species that are not directly dependent on sea ice may become increasingly important in predator diets if sea ice becomes unpredictable, with consequences for overall energetic intake of predator species (113).

The reduction in sea ice observed in the western Antarctic Peninsula has led to a shift from large diatoms, an important food source for krill, to smaller phytoplankton cells, e.g. cryptophytes (114). Salps, a group of gelatinous tunicates with a large water content (96%), can thrive under conditions of abundant smaller cells and warming temperatures (115, 116). There is DNA evidence that marine predators can feed on these gelatinous species. However, their quality as a food source (calorific value and protein content) is inferior to krill and fish, likely requiring a higher intake of salps to meet the energy needs of predators. While salps do overlap in distribution with krill (117), and have been observed under seasonal sea ice (118, 119), they are not a direct replacement in a food web because their reproductive and feeding modes are vastly different.

### Socio-economic and well-being impacts

The interconnectedness of sea ice within the Earth system is evidenced by the range of repercussions on human endeavor. Direct impacts include changes in sea ice affecting ice-navigation, near-coastal operations, fisheries, or immediate changes to structural ecosystem functions. Far-field effects include unseasonal or intense solid precipitation events at mid latitudes (120) and social and mental wellbeing.

#### Shipping and access

There are no commercial routing incentives for the Southern Ocean, with vessel activities largely centered around fisheries, tourism, research, and national operators (121). Nevertheless, the year-round reduction in sea-ice concentration and the shortening of sea-ice duration has already opened economic opportunities (122). The record for a vessel reaching the southernmost location on Earth was broken twice in recent years (123), which was only possible due to significantly reduced sea-ice concentration.

With reduced sea ice, the shipping pressures on the Antarctic continent will likely continue to increase, raising issues pertaining to marine biological invasions (124), fuel spills (125), and black carbon emissions (126).

Once in Antarctic waters, the itineraries of tourist vessels contain an element of flexibility. As such, there is a tendency to opportunistically exploit low sea-ice events to reach areas away from other vessels. Therefore we expect that even regional extreme low sea-ice events will lead to increased ship activities, especially near the Antarctic Peninsula.

Such expectation is corroborated by the Antarctic shipping network in high- and low-ice years (Fig. S8). While the temporal scope of the Automatic Identification System (AIS) data (2014–2018) considered in this study limits our ability to validate the findings, we compare the shipping network during the high sea-ice extent of 2014 with the one during the low sea-ice extent of 2017. In the low sea ice year, there were more gateway cities for ships visiting the Antarctic (189, compared to 154 in the high-ice case) and the number of visits increased (773, compared to 546 in the high-ice case).

In the absence of open water (i.e. secured ice-free access), operational support for Antarctic (near-) coastal stations and logistic hubs largely relies on stable landfast sea ice to provide access to the coast. A shorter sea-ice season will narrow the time window for over-ice resupplies. Less sea ice can also destabilize the ice-shelf edge (see Impacts on ice shelves section), increasing the risk of the shelf breaking up while a ship is parked for a resupply mission. This has already been reported, for example during the 2024 Norwegian Troll research station resupply cruise, when low sea ice concentrations exposed the ice shelf to waves and weather, leading to increased calving activity. Finding a safe offloading site was difficult and substantial delays ensued (127). Should this trend prevail, then national operators will need to prepare for alternative resupply methods, either by commencing their operational season earlier, by identifying alternative offloading sites, or by switching to more difficult over-water resupply methods.

#### Mental wellbeing and climate-anxiety

For decades, Antarctic sea ice was considered relatively “safe” by the research community, as its extent had stayed stable over the satellite—observation record, and it was receiving very little media attention. However, over the past decade Antarctic sea-ice extremes have started making it into media headlines (e.g. (128–130)). The exposure of the general public to Antarctic sea ice news can be quantified by trends in online searches on this topic. Globally people started searching the topic of “Antarctic sea ice” in 2007, with peaking interest at regular intervals, often timed with the sea ice minimum extent in February and maximum extent around September of each year (Fig. S9). There was a notable increase from September 2012 onwards (record high for Antarctic sea ice) in online searches of this topic culminating with a record high number of searches in July 2023 (Fig. S9), matching the extremely low winter sea ice extent that year. This increased exposure of the public to changes in Antarctic sea ice and, in particular, to decreasing sea ice extent in recent years, is very likely to fuel climate anxiety.

Climate-anxiety, also called “eco-anxiety” or “eco distress,” is the chronic fear and distress brought on by worrying about climate change and its impacts. Climate anxiety is well documented and has been studied over the past 15 years (e.g. (131, 132)). While the metrics in this research field are still being developed (133), early findings have identified young people and children (134, 135), indigenous communities (136), women (137), and climate scientists (138) as particularly prone to experiencing climate anxiety.

The cost of climate anxiety is not only the deteriorating mental health of individuals; it can also lead to a disengagement from the issue of climate change (139), potentially further reducing action to combat climate change.

## Conclusions

**Fig. 6.**
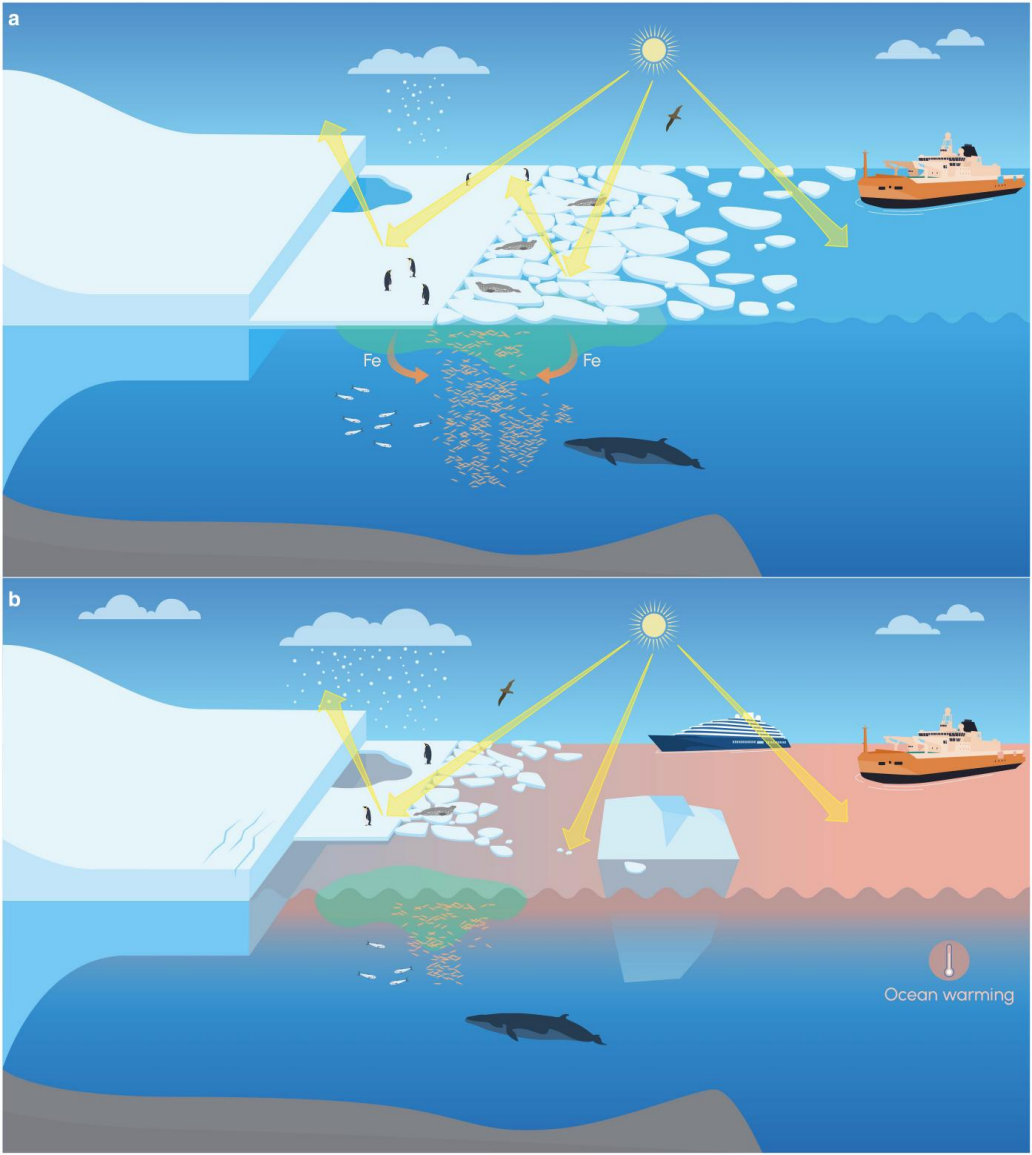
Extreme lows in sea ice induce many changes in the physical, ecological, and societal systems of Antarctica and the Southern Ocean. a) an average sea ice summer. b) an extreme low sea ice summer. In b), fast ice and pack ice have both retreated, ice floes within the pack ice are smaller, the surface albedo has decreased, and the surface ocean has warmed. There is also an increase in precipitation over the ice shelf. The reduction in sea ice has exposed the ice shelf to ocean waves, inducing fractures near the calving front, and leading to increased iceberg calving. There is a transition from sea ice associated productivity in a) to open ocean productivity in b) with a concomitant reduction in krill and silverfish biomass. In b), the seals are forced to seek shelter on smaller ice floes, while much of the landfast ice used by the penguins in a) has disappeared in b). In b), an opportunistic tourist vessel visits a region that was previously inaccessible due to ice cover.

We are only just beginning to understand the impacts of the recent extreme lows in sea-ice cover. These impacts are diverse, complex, and pervasive. They occur across physical, ecological, and societal systems within and around Antarctica (Fig. 6). Previous assessments of sea-ice extremes and their impacts have largely been limited to regional case studies (e.g. (18)). While the broad remit of this contribution necessarily limits the depth of our analysis, it highlights the large spatial scale of the impacts and the interdependent nature of the physical, biological, and societal systems under consideration. These significant impacts are of particular concern given the recent prevalence of low sea-ice summers, and projections of continued sea-ice loss.

The assessment of extremes in Antarctic sea ice is limited by a relatively short observational record that begins in 1978. However, recent variability is unprecedented in a 120-year reconstruction of sea-ice extent based on atmospheric data (140) and a fully independent 300 year reconstruction based on ice-core data (141).

Extreme reductions in sea-ice area occur simultaneously with extremes in multiple sea-ice metrics, highlighting the pervasive nature of these conditions within Antarctic sea ice.

Model experiments based on the 2016/2017 summer show a significant ocean response to extreme low sea-ice cover. There is a significant mixed layer warming in the sea-ice zone due to the reduced albedo, which persists for 3–4 consecutive summers, and in winter is expressed as reduced sea-ice growth. The persistent sea ice response is evident in volume but not in area, implying that the satellite sea-ice area record may under-represent the true response to extreme sea-ice conditions. The warming response to 2016/2017 persists for at least one summer longer than a simple linear estimate would suggest, implying that there is a threshold of sea-ice loss magnitude beyond which the ocean impacts are disproportionately increased. We have not identified that threshold, but it is clearly an important consideration for assessing the future impacts of sea-ice loss.

The change in surface-freshwater flux has an impact on Southern Ocean water masses. Freshening due to enhanced sea ice melt leads to an initial increase in the formation rate of Antarctic Intermediate Water, followed by a reduction in subsequent years. Antarctic Intermediate Water formation is an important mechanism for ocean-uptake of anthropogenic heat and carbon, suggesting that sustained future sea ice loss could impact the planet’s climate sensitivity.

Our analysis of observed iceberg calving rates shows a clear relationship between coastal exposure to damaging ocean swells and iceberg calving, as suggested by previous regional studies (14). A question for future research is whether the combination of swells and frontal melt could combine to drive a nonlinear increase in calving rates.

Observations from satellites and floats during the 2016/2017 and 2021/2022 extreme events highlight complex nonlinear sea-ice ocean interactions in the Southern Ocean biogeochemistry.

Antarctic wildlife may adapt behaviors to an extreme ice event, for example, by finding alternative foraging, breeding, or molting locations, or by prioritizing adult over offspring survival. Changes in the timing or extent of melt or formation of sea ice have species-specific impacts on the reproduction success of all Antarctic breeding species, or in the worst case, their survival. Recent events are unprecedented in their magnitude and spatial scale, and the capacity of species to adapt is unknown. Species migrating to foraging areas in the Southern Ocean may experience longer commutes if sea ice extent is reduced, but they may be less susceptible to population decreases than Antarctic breeding species. Conditions that reduce the amount of prey available to predators, e.g. an earlier break up of sea ice or a reduction in its area or extent, are critically relevant to all seabird, seal and whale species (Fig. 5).

Tourist vessels regularly visit Antarctica. Our results indicate that the vessel operators are able to opportunistically capitalize on extreme low sea ice coverage by increasing the number of ship visits, and by visiting new regions. Given that much of the environmental impact of these ships is currently localized, this potentially spreads these impacts over a broader region of the Southern Ocean and Antarctica.

In this study, we have largely focused on marine, cryosphere, ecological, and societal impacts, and have not mentioned the atmosphere. Modeling studies have shown that a reduction in the mean state of Antarctic sea ice extent drives a modest but significant weakening of the Southern Annular Mode (142, 143). A mean state reduction in Antarctic sea ice is expected to lead to changes in global temperature and rainfall patterns (144). However, in the context of extreme events, the timescale and strength of this teleconnection is uncertain. To our knowledge, so far only one study has considered the impact of isolated extreme events (145), and suggested that the 2016/2017 extreme summer may have contributed to a wavelike atmospheric response the following austral winter centered over the Pacific sector, reaching as far north as 30°S. In the context of societal impacts, this would potentially affect weather in Australia and New Zealand, and indicates that further research is necessary to ascertain whether there is a detectable signal of sea ice extremes in these population centers. We have also not considered changes in ice sheet surface mass balance. Historical changes to Antarctic surface mass balance have been both small and highly uncertain, but recent work shows that sea ice loss increases the atmospheric moisture transport to the Antarctic continent (146), increasing precipitation and leading to positive changes to the surface mass balance. However, this is predicated on the assumption that precipitation is expressed as snow rather than rainfall. As the continent warms (in part due to sea ice loss (147)), rainfall is expected to become a higher fraction of precipitation (148). The complicated interactions between ocean, sea ice, atmosphere, and ice sheet are an important factor in predicting Antarctica’s future.

### Future priorities

To fully understand the impact of Antarctic sea ice extremes, we require significant advances in both our understanding of the physical and ecological systems, and in our ability to observe them. The single most critical measurement that we currently lack is circumpolar sea-ice thickness. Without reliable, year-round, long-term measurements of sea-ice thickness, we are unable to assess the multiyear persistence of sea ice extremes, or identify regions where sea ice is thinning and likely to retreat more rapidly.

Long-term observing is the only way to obtain the data required to assess the physical, ecological, and societal impacts of extreme events in Antarctic sea ice and to link these to changes in the global Earth system. Without a well-observed baseline, it is not possible to assess changes. Many of our current observations are insufficient to assess the response of Antarctica and the Southern Ocean to extreme events. This assessment requires higher temporal and spatial resolution datasets, as well as broad spatial coverage. Remote sensing observations provide circumpolar data on many critical components of these systems, but satellites are unable to measure many crucial variables, especially snow and sea-ice thickness. It is therefore vital that we maintain and expand our in situ observational capabilities (149, 150). Improving our understanding of the rapid sea ice and ocean changes currently occurring in the Southern Ocean requires year-round measurements of temperature and salinity under the sea ice. In addition, understanding the impacts of sea ice changes on primary productivity, carbon export, and higher trophic levels requires integrated measurements of physical and biogeochemical variables in the seasonal sea ice zone. These observations would also allow for robust evaluation of modeling results, such as those presented in Oceanographic impacts section. To date, observational campaigns have focused more on West Antarctica, and in particular around the Antarctic Peninsula. However, where data are available, impacts have been observed in East Antarctica as well, highlighting the urgent need for circumpolar observations.

These are the priorities for a scientific community that seeks to understand the impacts of sea ice extremes. As discussed above, there are clear indications that Antarctica and the Southern Ocean are already changing, and work is urgently required to understand the mechanisms controlling these changes. However, climate projections indicate that continued greenhouse gas emissions will accelerate these changes (151). Our initial analysis shows that there are far reaching negative impacts caused by sea ice loss. Therefore, a society that seeks to conserve and preserve the physical environment and ecosystems of Antarctica and the Southern Ocean must prioritize an immediate and sustained transition to net zero greenhouse gas emissions.

## Materials and methods

### Observed changes in sea ice

Our method for ranking extreme years is based on the variables that represent summertime parameters and have an observational time series back to 1979 (i.e. summertime minimum area, duration of open water and coastal exposure) (see Supporting Information for further details). Using this method, we confirm that the austral summers of 2011/2012, 2012/2013, 2013/2014, and 2014/2015 are “high sea-ice years,” and 1992/1993, 2016/2017, 2021/2022, and 2022/2023 are “low sea-ice years” in line with previous studies (e.g. Ref. (1)).

### Oceanographic impacts

We use the ACCESS-OM2-025 coupled ocean-sea ice model to run a control simulation that faithfully reproduces the observed sea ice area record (152) and a perturbation experiment in which we take the ocean and sea ice state from the end of 2014 and skip the model forward 2 years to the beginning of 2017. Because this model uses a data atmosphere from reanalysis, we could avoid the Southern Ocean biases commonly found in fully coupled ocean—sea ice—atmosphere models. Using the ACCESS-OM2-025, modeling framework allows us to assess the impact of the dramatic sea ice loss in 2015 and 2016 on the state of the coupled ocean—sea ice system.

### Impacts on ice shelves

To assess the impact of sea ice extremes on iceberg calving rates, we compare summer (December–January–February) sea ice area to ice shelf exposure and iceberg calving observations. We use a previously calculated dataset of the number of days each ice shelf is exposed to ocean swell (ice-shelf exposure, (44)), and a 15-year circum-Antarctic dataset of iceberg calving events derived from continuous satellite observations (153).

### Impacts on primary productivity

We tested the relationship between sea ice loss and primary productivity for the 2016/2017 and 2021/2022 sea-ice events using stellite remote sensing and BGC-Argo floats. Details on the use of satellite remote sensing and BGC-Argo float data in polar regions are given in the Supporting Information, including quality control and methods limitations.

## Supplementary Material

pgaf164_Supplementary_Data

## Data Availability

Observational datasets are available from the public repositories described in the relevant references. Due to size (>100 GB), the ocean and sea ice modeling data used to derive a number of the results in this manuscript are not available in a persistent publicly accessible location. They are currently stored on the servers at the Australian National Computational Infrastructure. Modeling data can be shared upon request by creating an account at https://my.nci.org.au/.
