# Supplementary material for: Impacts of Antarctic summer sea-ice extremes
Source: PNAS Nexus. 2025 Jul 1;4(7):pgaf164. doi: 10.1093/pnasnexus/pgaf164 (PMC12210234; doi:10.1093/pnasnexus/pgaf164)
Supplement: pgaf164_Supplementary_Data [file pgaf164_supplementary_data.pdf]

Supplementary Information for
Impacts of Antarctic Summer Sea-Ice Extremes

Edward W. Doddridge,[a] Will Hobbs,[a,b] Matthis Auger,[c,d] Philip W. Boyd,[a]
Sean M. T. Chua,[e,a] Sue Cook,[a] Stuart Corney,[c,a] Louise Emmerson,[e]
Alexander D. Fraser,[a,c] Petra Heil,[e,a] Nat Kelly,[e] Delphine Lannuzel,[c,d,a] Xueke Li,[f,g]
Guillaume Liniger,[b,h] Robert A. Massom,[e,a,d] Amelie Meyer,[c,b] Phillip Reid,[i,a]
Colin Southwell,[e] Paul Spence,[a,c,d,j] Anton Steketee,[e,a,k] Kerrie M. Swadling,[c,a]
Nathan Teder,[l] Barbara Wienecke,[e] Pat Wongpan,[a] and Kaihe Yamazaki[c,d]

a Australian Antarctic Program Partnership, Institute for Marine and Antarctic Studies, University of Tasmania, nipaluna / Hobart, Tasmania, Australia
b ARC Centre of Excellence for Climate Extremes, University of Tasmania, nipaluna / Hobart, Tasmania, Australia,
c Institute for Marine and Antarctic Studies, University of Tasmania, nipaluna / Hobart, Tasmania, Australia
d The Australian Centre for Excellence in Antarctic Science, University of Tasmania, nipaluna / Hobart, Australia
e Australian Antarctic Division, Kingston, Tasmania, Australia
f Institute at Brown for Environment and Society, Brown University, Providence, Rhode Island, USA
g Currently at Department of Earth & Environmental Science, University of Pennsylvania, Philadelphia, PA, USA
h Monterey Bay Aquarium Research Institute, Moss Landing, California, USA
i Australian Bureau of Meteorology, nipaluna / Hobart, Tasmania, Australia
j The Australian Centre for Excellence for Weather of the 21st Century, Institute for Marine and Antarctic Studies, University of Tasmania, nipaluna / Hobart, Tasmania, Australia
k Currently at Australian Earth System Simulator (ACCESS-NRI), Australian National University, Canberra, Australia
l University of Adelaide, Adelaide, South Australia, Australia

Edward W. Doddridge
Email: Edward.doddridge@utas.edu.au

This PDF file includes:

- Supplementary text
- Figures S1 to S9
- Tables S1 to S2
- SI References

Supplementary Information Text

Detailed Methods

Sea Ice. We used daily sea ice concentration calculated using the NASA-Team algorithm from the National Snow and Ice Data Center (Meier et al., 2021a, 2021b) for the length of the satellite data record (1979-2023). Total circum-Antarctic sea ice area was calculated by removing concentrations less than 15% and then calculating the area-weighted sum of concentration. We used the 5-day rolling mean of the circum-Antarctic daily sea ice area to find the annual sea ice minima and maxima.

To investigate changes to sea ice seasonality which may co-occur with variability in sea ice area, we calculated open water duration, derived from sea ice advance and retreat. Following Stammerjohn et al. (2008), an annual window from mid-February (year day 46) to the next mid-February (year day 410/411) is defined to describe the year between two sea-ice minima. Day of advance is calculated as the first day that the five-day rolling average, i.e. average of the last 5 days, of sea ice concentration is equal to or greater than 15%. Day of retreat is the last day that the five-day rolling average of sea ice concentration drops below 15%. Instead of the typical measure of the length of a sea ice season, i.e. the duration between advance and subsequent retreat, we focused on the summer open water duration, calculated as the duration between retreat and the subsequent advance. If advance occurred after September 15 (or retreat occurred before September 15) in a grid cell, its advance/retreat value was set to a default value of September 15 in order to avoid open water duration values of longer than one year. Ocean grid cells where no sea ice was present were given an open water duration of 365/366 days. The time series of open water duration was then calculated as the area-weighted mean for all ocean grid cells south of 55°S. Each year was labelled based on the second year, e.g. 2021/2022 austral summer was labelled as 2022.

We obtained sea-ice thickness estimates from the recently-updated Version 3 of the ESA Climate Change Initiative Antarctic sea-ice thickness dataset, which estimates thickness from altimetric measurements of sea-ice freeboard from Envisat and CryoSat2. In Version 3, Envisat data have been improved and validated during the instrumental overlap with CryoSat2 (Paul et al., 2021) We present annual May-Oct means as we have greater confidence in sea-ice thickness measurements during the growth season.

Coastal exposure length is based on the definition within (Reid and Massom, 2022), and is the detection of a lack of sea ice directly offshore from the Antarctic coastline. Coastal exposure length is the length of coastline with no sea-ice adjacent, excluding polynyas, and has a value between 0 and ~17,850 km. Values cited within Figure 1 are the time-integral of coastal exposure length between sea-ice maxima, calculated as the sum of the daily coastal exposure length between consecutive 15ths of September. The years are labelled based on the second year, e.g. 2021/2022 coastal exposure days is labelled as 2022.

Landfast ice extent at the time of minimum extent (i.e., in early-mid March) is taken from Fraser et al. (2021) and Fraser et al. (2023). The mapping of landfast ice is semi-automated for 2000-2018 based on cloud-free composite imagery and a persistence-of-edges approach detailed in Fraser et al. (2020). Landfast ice maps for 2019-2022 are manually delineated using the methodology of Fraser et al. (2010).

To determine years with extreme high and low sea ice for further analysis, we ranked then summed three annual datasets: annual sea ice minimum area, open water duration and coastal exposure days. The open water duration and coastal exposure were inverted before ranking, so that high ranks for all three datasets indicate more sea ice, and vice-versa. The ranks from the three datasets were then summed. Following the marine heatwave community (e.g., Hobday et al., 2016), we employ upper and lower deciles to indicate extreme years (i.e. four extreme high and low years from a 44-year record).

Ocean. We use the Australian Community Climate and Earth System Simulator Ocean Model (ACCESS-OM2) to model the impact of extreme low summer sea ice. This model links a sea ice model (CICE5), an ocean model (MOM5), and a data atmosphere (JRA-55do). The use of a data atmosphere allows us to avoid the Southern Ocean biases that are common in fully coupled climate models.

Our perturbation simulation takes the model state at the end of 2014 and skips two years' worth of atmospheric data. This produces a model in which the atmospheric state from the beginning of 2017 onwards is applied to an ocean - sea ice model that has not undergone the striking loss of sea that occurred during 2015 and 2016. We define the anomalies as the difference between the control and perturbation simulations.

We are unable to assess the impact of more recent sea ice extremes, because we require four years of atmospheric data after the sea ice extreme to run the ocean model. These data do not yet exist for the more recent extreme summers we identified (2021/22 and 2022/23).

Ice Shelves. The correlations between sea ice area, exposure days, and iceberg calving were performed in multiple sectors as well as for the circumpolar total (Table S1).

Primary Productivity.
Satellite remote sensing - Ocean color. Previous studies highlighted the role of sea ice cover on marine productivity, where decline in sea ice cover and extent was associated with stronger biological production in the Arctic (Arrigo et al., 2008; Arrigo and Van Dijken, 2015). Therefore, understanding how this relationship behaves during extreme events is important, as such a relationship has further consequences on the carbon cycle.

To investigate the long-term changes in surface phytoplankton biomass, we used Level-3 satellite surface chlorophyll-*a* concentration with temporal and spatial resolution of 8 days and 0.04° (~4 km) respectively from the European Space Agency (ESA) GlobColour project (https://www.globcolour.info/). We used the standard GSM model Case 1 water (CHL1, open water, where phytoplankton is assumed to drive optical properties, therefore, waters that can be described as a function of chlorophyll-*a* concentration, a proxy for phytoplankton biomass) merged products consisting of the Sea-viewing Wide Field-of-view (SeaWiFS), Medium Resolution Imaging Spectrometer (MERIS), Moderate Resolution Imaging Spectroradiometer (MODIS-A) and Visible Infrared Imaging Suite sensors (VIIRS). Before merging, SeaWiFs, MODIS-A and VIIRS were reprocessed following the NASA R2018 algorithm, while MERIS followed the ESA 3rd reprocessing. Documentation regarding the sensors can be found at https://www.globcolour.info/CDR_Docs/GlobCOLOUR_PUG.pdf/

In the Southern Ocean, satellite remote sensing of ocean color faces several challenges influencing the data quality. Firstly, the large solar zenith angles can affect light reflectance, potentially leading to an underestimation of in situ ocean chlorophyll-*a* (Sirjacobs et al., 2011; Wang, 2003). Other atmospheric features such as aerosols and clouds can decrease spatial coverage, leading to potential biases and lower data quality (Pereira and Garcia, 2018; Sirjacobs et al., 2011).

Because we delve into the relationship between surface chlorophyll-*a* and sea ice, it is important to acknowledge the impact sea ice has on satellite ocean color data. Firstly, much like clouds, the presence of sea ice reduces data to the open water period. Sea ice also impacts the reflectance used to calculate chlorophyll-*a* (Pereira and Garcia, 2018), as sea-ice meltwaters release particles which backscatter more light, thereby modifying the reflectance (Dierssen et al., 2002). Some algorithms were developed specifically for the Southern Ocean (Johnson et al., 2013; Kahru and Mitchell, 2010), but have been found to overestimate chlorophyll-*a* in the sea-ice zone (Haëntjens et al., 2017), which is within our area of interest, while the Ocean Color Index (OCI, method used for our satellite data (Hu et al., 2012)) performs well in the circumpolar Southern Ocean. The use of satellite remote sensing and merged products from different sensors has become a robust tool to investigate phytoplankton blooms and associated phenology in recent years (Ardyna et al., 2017; Del Castillo et al., 2019; Kauko et al., 2021; Liniger et al., 2020; Thomalla et al., 2023) and is therefore the method of choice in our study.

We computed two climatologies, both of which use the 8-day data. One is used for Figure 5 in the main article, and the other from each 8-day period for the comparison between the BGC-Argo data and satellite data. For Figure 5, we averaged chlorophyll-*a* (from 8-day data) over the corresponding open water period for each pixel and each year (Both chlorophyll-*a* and sea-ice concentration datasets were interpolated on to the same 25km grid). The average chlorophyll-*a* for a given pixel

(2004 to 2018) represents the climatology that we used as a baseline to compare with the 2021/22 event.

For the comparison between satellite chlorophyll-*a* and the BGC-Argo float, we use a similar method. However, instead of averaging the chlorophyll-*a* pixels over the open water period for each year, we take a given chlorophyll-*a* concentration 8-day period and average from 2004 to 2018. For example, we took all 8-day periods starting January 1st and averaged them all to obtain a climatological baseline for that 8-day period. We repeated this for the forty-six 8-day periods in a year. We then compared our 8-day baseline for the closest 8-day period (week starting February 26th) to the time where the float took the #220 salinity profile (February 27th). Due to the patchy nature of 8-day data, no chlorophyll-*a* data was available in the closest pixel to our float location when profile #220 was taken. We therefore performed an average on a 5x5 grid point box from the closest location for both the climatology and the late February 2022 data to investigate the change.

Satellite remote sensing - Sea ice. The sea-ice phenology (advance, retreat, duration) was computed following Stammerjohn et al. (2008), using a 15% sea-ice concentration threshold. For each pixel, Advance was calculated to start on the 46th day of the year (15th Feb). To advance the pixel needs to have sea-ice concentration greater than 15% for a run of 5 days or more. To retreat, the pixel needs to have concentration less than or equal to 15% for 5 days or more. The duration was defined as the time between the day of advance and day of retreat. Conversely, the open water duration was defined as the time between the first day of retreat and first day of advance. The sea-ice data used are from Nimbus-7 SMMR and DMSP SSM/SSMIS Passive microwave data with a 25 km resolution downloaded from the National Snow and Ice Data Center (NSDIC) website (https://nsidc.org/data/nsidc-0051/versions/2#anchor-2).

BGC-Argo float. As mentioned above and in the main text, ocean color satellite remote sensing cannot see through clouds and sea ice, and cannot resolve oceanographic processes at depth. To overcome this, we used BioGeoChemical (BGC)-Argo floats to investigate in situ changes in salinity, temperature and chlorophyll-*a* in the water column during the 2016/17 and 2021/22 sea-ice low events. We selected floats present in the sea-ice zone/marginal ice zone at the time of both events that showed at least 1 annual cycle prior to each event. The floats were equipped with salinity, temperature and chlorophyll-*a* fluorescence sensors. We downloaded “adjusted” data from floats corresponding to these criteria using the One-Argo mat toolbox (Frenzel et al., 2022), from floats 7900673, 5904397 and 5904180. Temperature, salinity and pressure data were rigorously quality controlled (Bittig et al., 2019; Schmechtig et al., 2023). Using “adjusted” data for all variables takes into account the regular adjustments made to their calibration post deployment. Extensive explanation on chlorophyll-*a* quality control can be found in Schmechtig et al. (2023). This includes the primary data adjustment process for dark estimation, non-photochemical quenching (NPQ) and slope correction, and further correction taking into account particles impacting fluorescence that are not chlorophyll-*a* (including particles released by sea-ice melt). We only kept data showing quality flags of 1, 2, 5 and 8. These flags correspond to (1) good data, (2) probably good, (5) value changes and (8) good data interpolated on the same pressure level as other variables. On top of that, we also corrected chlorophyll-*a* data for potential NPQ following Xing et al. (2018).

Matching up satellite and float data. For the 2021/22 event, no chlorophyll-*a* data was available from the BGC-Argo float. Therefore, to match up chlorophyll-*a* satellite and changes in salinity from the BGC-Argo float, we used the time at which salinity profiles from the floats were taken. We evaluated salinity in October 2021 (start of sea-ice retreat, profile #205), noting that no satellite chlorophyll-*a* was available. We therefore focused on March 2022 (full melt, profile #220) to match up the closest pixel of anomalous chlorophyll-*a* data for the same time period. Because daily satellite data show significant spatial gaps, we performed the match-up using the same 8 day averaged time period data described in the satellite remote sensing.

Marine predators and their prey: Expert assessment of impacts on higher trophic levels. Recent extreme events in Antarctica had not been predicted (Massonnet et al., 2023). Thus, no dedicated field campaigns were undertaken to study the impact of these events on higher trophic levels. Therefore, we assessed potential impacts of extreme events on higher trophic levels by applying a conceptual approach based on expert judgement, and used several criteria to make a qualitative prediction of the ecological vulnerability of several Antarctic species. The effect of each

kind of extreme event was assessed against species vulnerability with regard to impact on habitat, food supply, adult survival, breeding success, survival of juveniles and population trajectory. Five competent, experienced experts with relevant expert knowledge of species biology, ecology, and the functional mechanisms and causal relationships with their environments independently considered the impacts of extreme events.

Next individual assessments were compared and discussed, and then assigned a level of confidence when agreement on the direction of an impact was reached. Depending on the quality of available data or level of agreement among the experts, confidence scores were assigned. High confidence indicates high consensus and/or access to adequate data; where experts agreed that multiple processes could act in different directions a low confidence score is indicated. Where data were clearly insufficient and outcomes unclear, the effect of impacts was indicated as unknown. These still delivered important information as they highlight knowledge gaps. Given the lack of sufficient information and high-quality data (extreme events have been rare and not every aspect of them has been investigated in detail), this was an informal approach based on experience, intuition and logic. This qualitative assessment provides a baseline against which future events can be compared and the assessment updated.

Socio-economic and well-being impacts: Online search interest for “Antarctic sea ice”. Google Trends is a tool that can be used to quantify online searches for particular topics over a defined region and time period. According to Google Trends, global online searches on the topic of ‘Antarctic sea ice’ over the period of 2004-2024 started in 2007 with a marked increase from September 2012 onwards, and a maximum peak of interest in July 2023 (Figure S9} and Google Trends (2024). The interest values do not represent absolute search volume. Instead they are normalized, and then indexed on a scale from 1-100, where 100 is the maximum search interest for the period selected. The normalization means that we are not looking at the total number of searches but instead at the percentage of searches for that topic, here ‘Antarctic sea ice’, as a proportion of all searches over the selected period. Related ‘rising’ online searches with the biggest increase in volume for the same time period included the search terms ‘Antarctic’, ‘Antarctic ice’, ‘Antarctic ice extent’ and ‘Antarctic sea ice extent’, all qualifying as ‘Breakout’ online searches (not shown). Breakout searches means that these searches had an enormous increase and are formally identified as when search terms grow by more than 5000%.

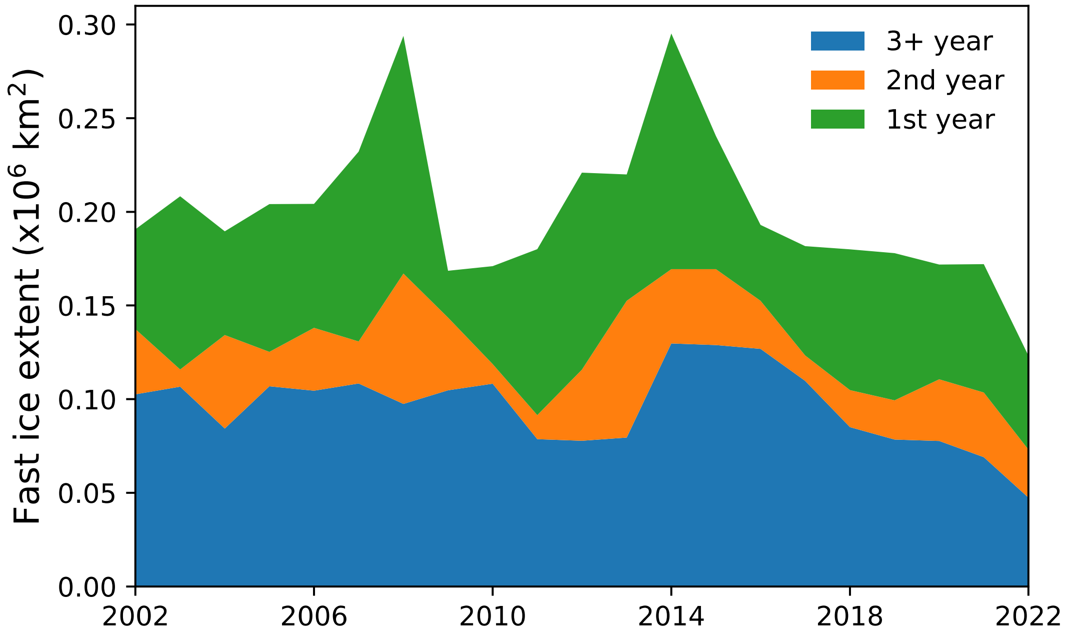

Fig. S1. Satellite-derived circum-Antarctic annual minimum total landfast ice extent (occurring in early March) by ice age for the years 2004-2022. Note the substantial loss of 3+ year-old landfast ice since 2020.

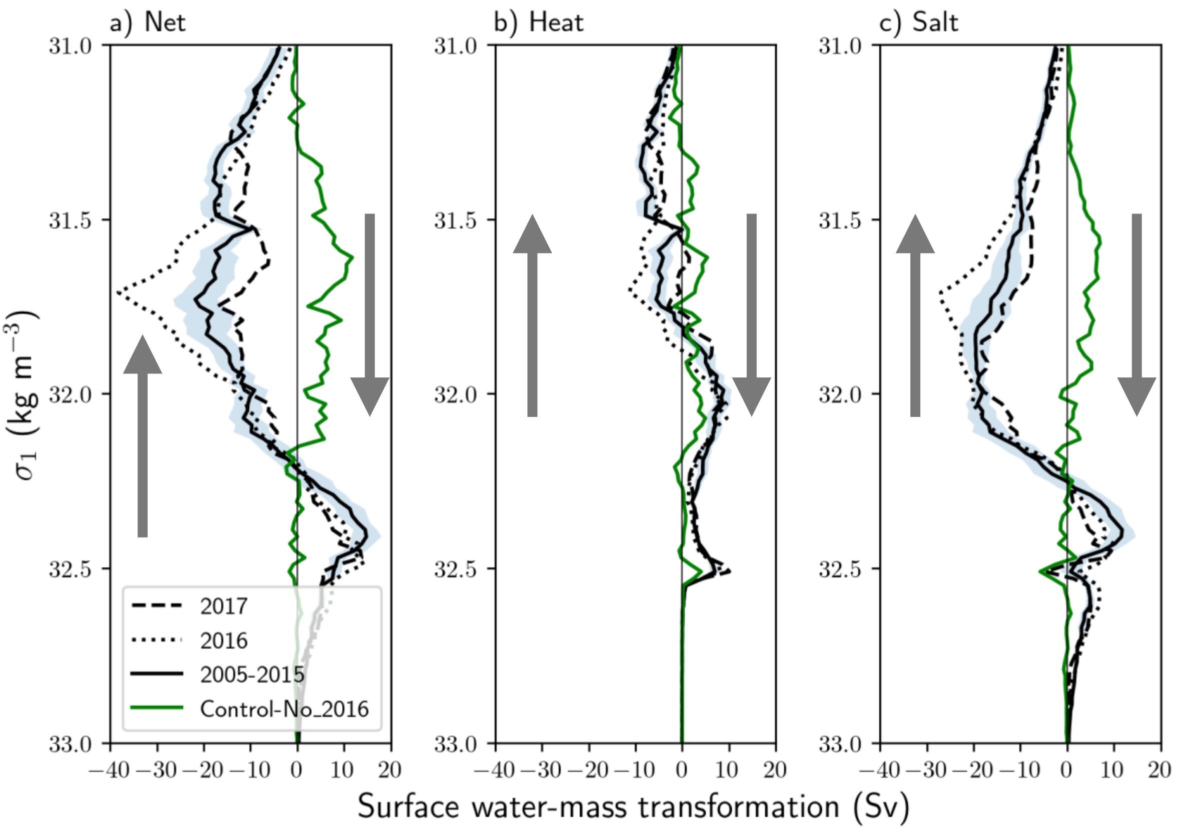

Fig. S2. Surface water-mass transformation (Sv) south of 55°S in the ACCESS-OM2-025 model. The net (a) transformation is decomposed into heat (b) and salt (c) components and binned in density classes referenced to 1000 db. The solid black lines are 2005-15 averages with blue shading showing a one standard deviation annual mean range. The black dotted and dashed lines are the 2016 and 2017 annual averages from the Control simulation. In general terms, the model transformation rates depict the lightening of Circumpolar Deep Water into Antarctic Intermediate Water within the 31.0-32.2 kg m^{-3} range and the densification of Circumpolar Deep Water into Antarctic Bottom Water within the 32.2-32.8 kg m^{-3} range. We can see that during 2016 substantially more water was transformed into Antarctic Intermediate Water (dotted line, a) and that most of this effect was due to changes in salinity (dotted line, c); extra sea ice melted in 2016 and produced more Antarctic Intermediate Water. However, in 2017 there was a reduction; the record low summer in 2017 meant there was less sea ice available to melt and create Antarctic Intermediate Water. The green lines are anomalies in 2017 of Control minus the No_2016 simulation showing that the extreme sea ice low lead to a reduction in the formation rate of Antarctic Intermediate Water in 2017. A negative transformation represents a lightening of water, as indicated by the grey arrows.

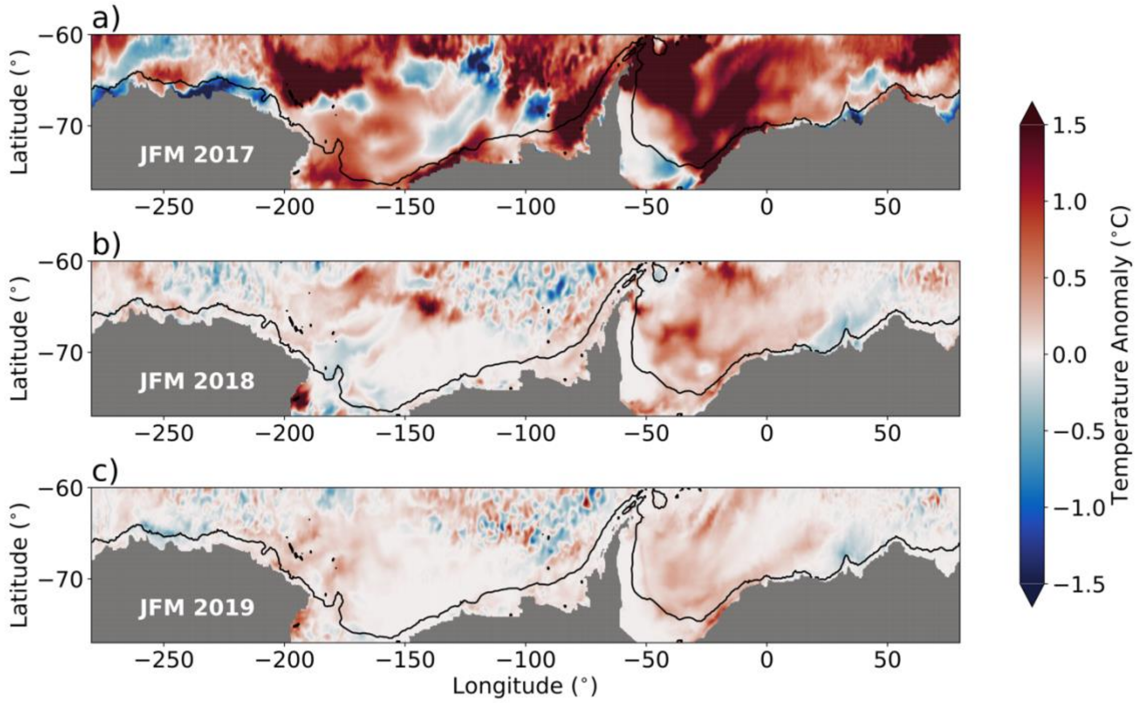

Fig. S3. Upper layer (0-50m) summer temperature response to the extreme sea ice loss in 2015 and 2016 from the ACCESS-OM2-025 model. 2017 (a), 2018 (b), and 2019 (c) summer (JFM; January February March) temperature anomalies, showing that the zonal-mean multiyear memory demonstrated in Figure 2 is a widespread phenomenon both on and off the continental shelf. Anomalies are the Control minus the No_2016 temperature. The black contour is the 1000m isobath.

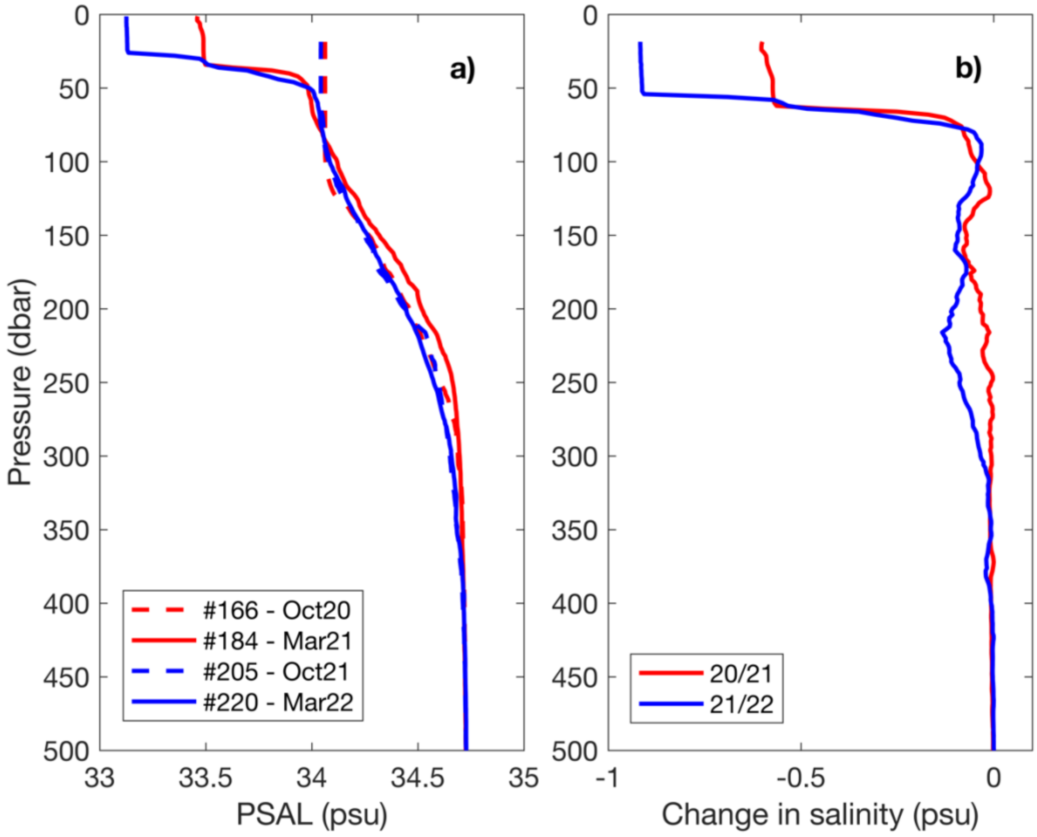

Fig. S4. (a) Salinity profiles for the four profiles investigated in this study. (b) Change in salinity between #184 and #166 (red, season 2020/21) and #220 and #205 (blue, season 2021/22).

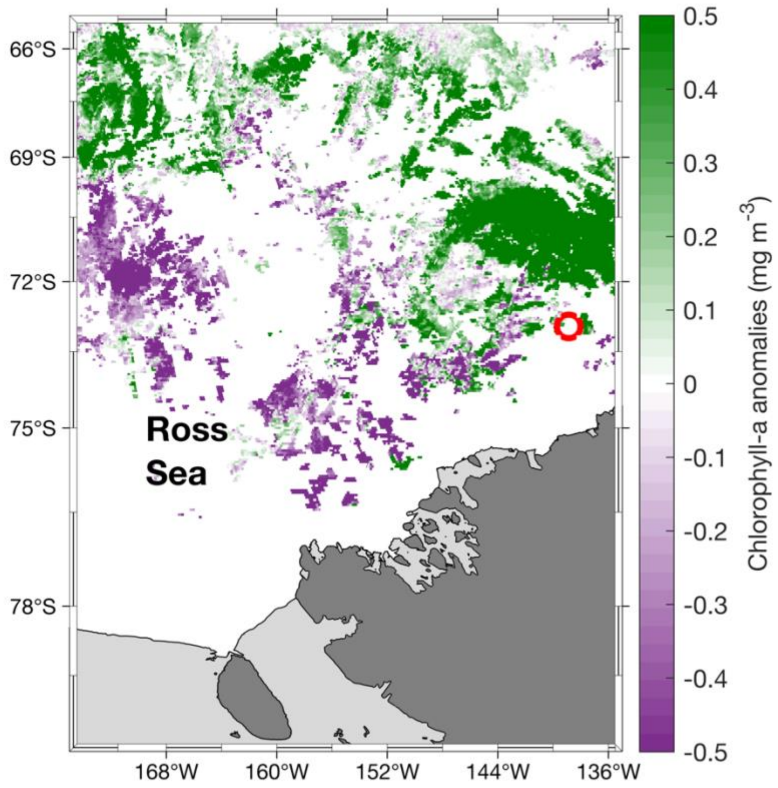

Fig. S5. 2021/22 8-day chlorophyll-*a* anomalies for week starting February 26th with #220 (February 27th 2022) profile location (red circle) in the Ross Sea during the 2021/22 season.

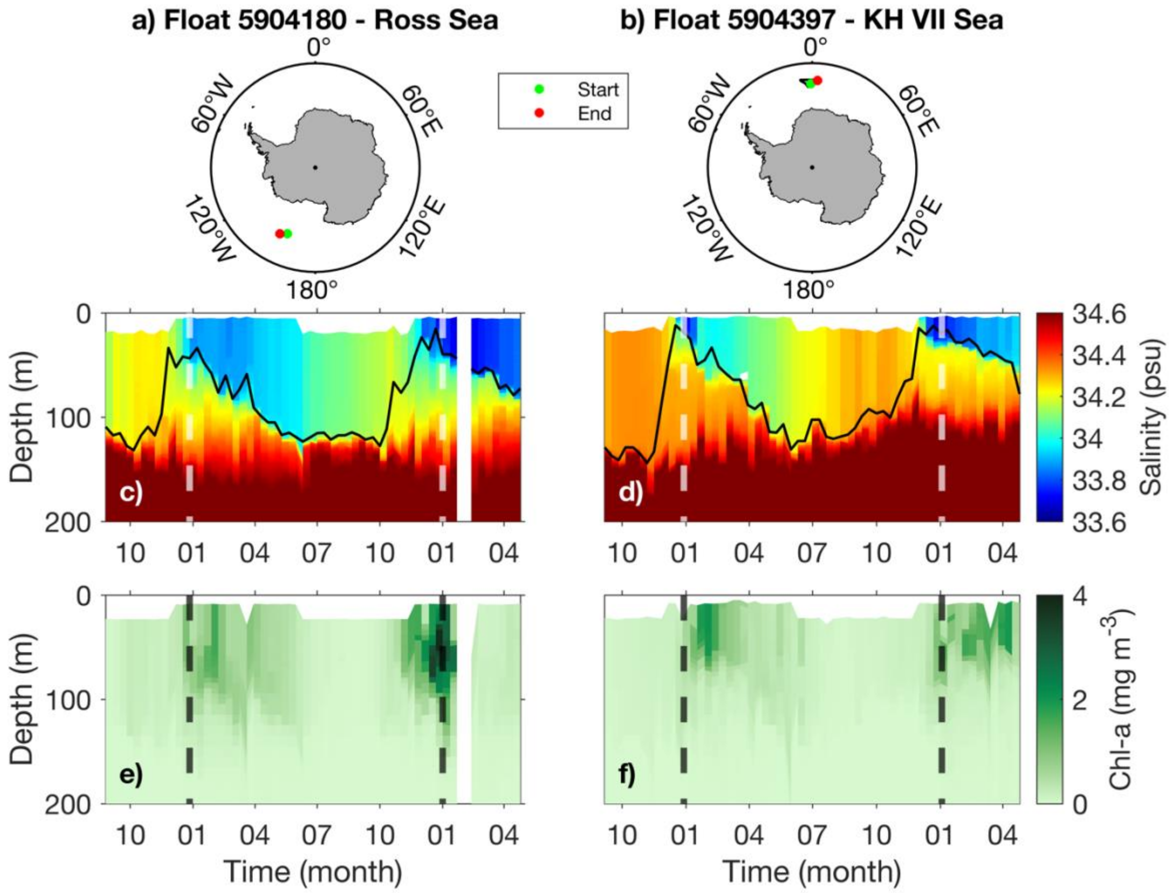

Fig. S6. BGC-Argo float #5904180 and #5904397 locations from September 2015 to 2017 in (a) the Ross Sea and (b) KH VII Sea. Section plots show the change in salinity and chlorophyll-*a* (chl-a) for (c-e) the Ross Sea and (d-f) King Haakon VII Sea. The thick white and black dashed lines represent the start of calendar years 2016 and 2017.

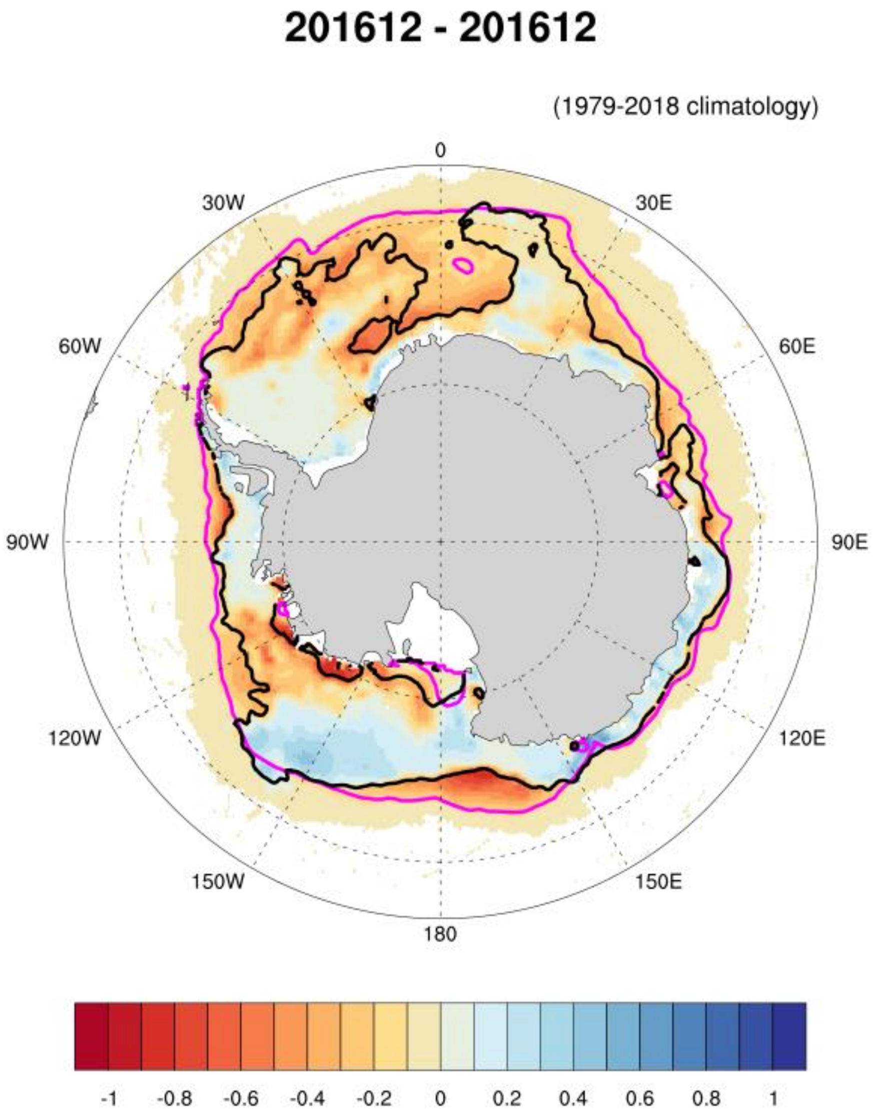

Fig. S7. December 2016 sea ice concentration anomaly (with respect to 2004-2018 climatology, from NSIDC Climate Data Record (Meier et al., 2021a)). Magenta line shows the climatological ice edge (i.e., the 15% sea ice concentration isoline); black line is the December 2016 ice edge.

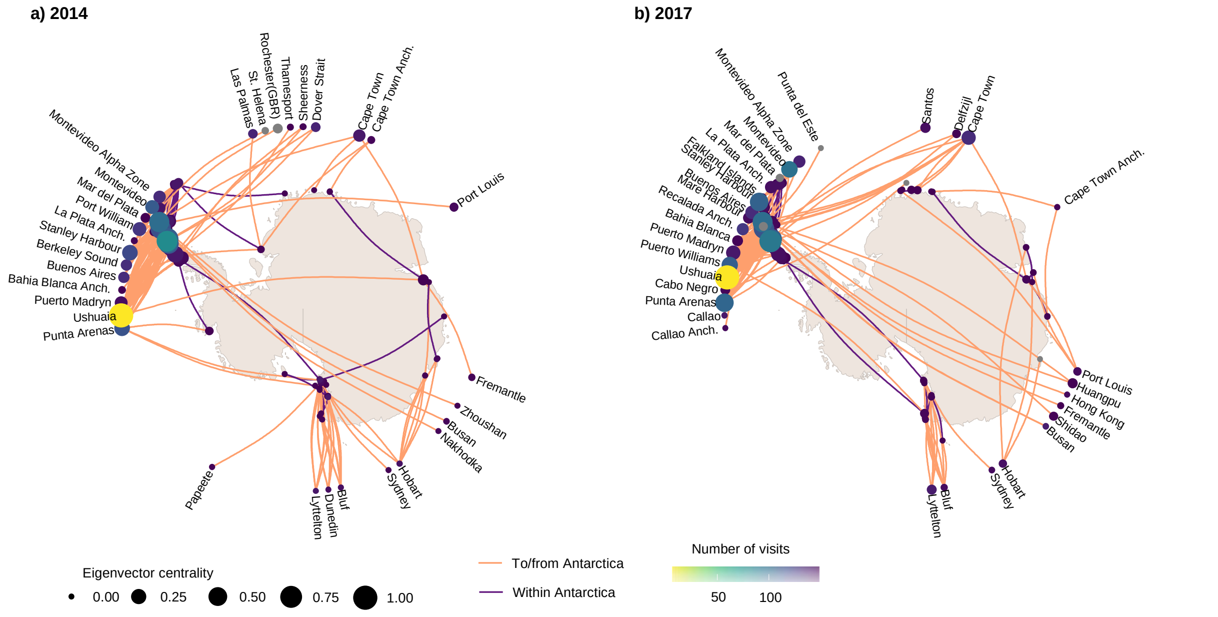

Fig. S8. The port-to-port shipping network of summer (January - March 2014 and 2017) visits to Antarctica, following both a high sea-ice extent year and a low sea-ice extent year, respectively. The maps are modified from Figure 2 of (McCarthy et al., 2022). The lines on the map depict connections between locations but do not illustrate the specific path of travel. The color of each circle indicates the frequency of visits to a particular port, while the size of the circle represents eigenvector centrality. Eigenvector centrality measures the relative importance of ports in the network based on their connectivity to other highly ranked ports.

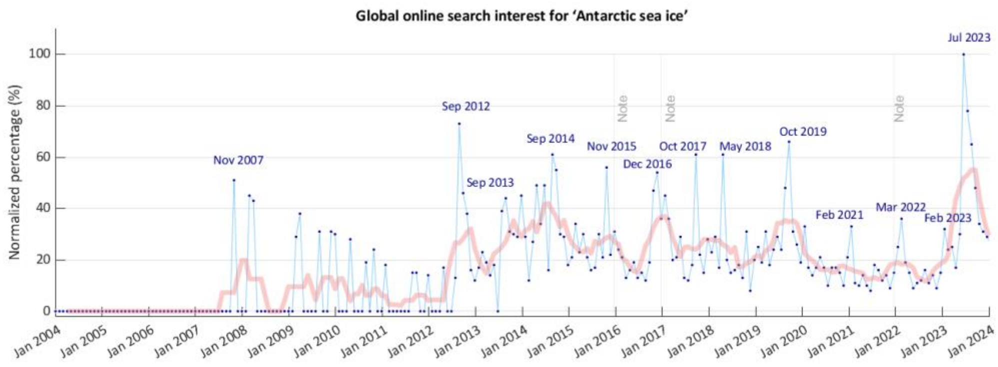

Fig. S9. Time series of the normalized percentage of global online search interest for 'Antarctic sea ice' over the 2004-2024 period based on Google Trends data. Monthly data in blue and 6-month centred rolling average in red. Dates with increased interest are labelled. Vertical grey 'Note' lines indicate when improvements in the data collection were implemented.

Table S1. Results from sector-by-sector and continent-wide Spearman's rank correlation comparing summer-average sea ice area with number of days of coastal exposure and total iceberg count per year. We use Spearman's rank correlation as the number of icebergs is not expected to be normally distributed due to the self-organized criticality of calving processes (Åström et al., 2014). Statistically significant relationships (95% confidence) are highlighted in bold font.

Summertime average sea ice area in:	Exposure days			Iceberg count		
	ρ	p	slope	ρ	p	slope
King Haakon VII Sea	**-0.58**	**0.03**	**$-3.8*10^{-6}$**	0.02	0.94	$-1.3*10^{-6}$
East Antarctica	-0.42	0.12	$-4.8*10^{-6}$	**-0.77**	**$7.2*10^{-4}$**	**$-2.7*10^{-5}$**
Ross Sea	-0.30	0.27	$-4.9*10^{-6}$	**-0.75**	**0.01**	**$-8.8*10^{-6}$**
Amundsen and Bellingshausen Seas	0.01	0.96	$-2.2*10^{-8}$	-0.33	0.22	$-2*10^{-5}$
Weddell Sea	**-0.79**	**$8.2*10^{-4}$**	**$-7.9*10^{-6}$**	-0.46	0.08	$-4.9*10^{-6}$
Circumpolar	**-0.65**	**$8*10^{-3}$**	**$-4.5*10^{-6}$**	**-0.69**	**$4.1*10^{-3}$**	**$-6.2*10^{-5}$**

Table S2. Estimates of added dissolved iron and chlorophyll-*a* (chl-a) from sea-ice meltwaters for BGC Argo floats operating in Ross Sea. Profiles #166 and #184 are in October 2020 and March 2021 respectively, while #205 and #220 are in October 2021 and March 2022. Profiles #205 and #166 show the same values and are therefore grouped.

ARGO profiles Float #790063	Meltwater fraction	Min dFe added (nM)	Max dFe added (nM)	Mean dFe added (nM) ± stdev	Mean increase chlorophyll-*a* (mg m^{-3}) ± stdev
205 & 166	0.02	0.01	0.74	0.2 ± 0.3	0.16 ± 0.24
220	0.043	0.03	1.59	0.42 ± 0.65	0.34 ± 0.53
184	0.037	0.03	1.38	0.37 ± 0.56	0.30 ± 0.45

SI References

Ardyna, M., Claustre, H., Sallée, J., D'Ovidio, F., Gentili, B., Van Dijken, G., D'Ortenzio, F., Arrigo, K.R., 2017. Delineating environmental control of phytoplankton biomass and phenology in the Southern Ocean. Geophys. Res. Lett. 44, 5016–5024. https://doi.org/10.1002/2016GL072428

Arrigo, K.R., Van Dijken, G., Pabi, S., 2008. Impact of a shrinking Arctic ice cover on marine primary production. Geophys. Res. Lett. 35, 2008GL035028. https://doi.org/10.1029/2008GL035028

Arrigo, K.R., Van Dijken, G.L., 2015. Continued increases in Arctic Ocean primary production. Prog. Oceanogr. 136, 60–70. https://doi.org/10.1016/j.pocean.2015.05.002

Åström, J.A., Vallot, D., Schäfer, M., Welty, E.Z., O'Neel, S., Bartholomaus, T., Liu, Y., Riikilä, T., Zwinger, T., Timonen, J., others, 2014. Termini of calving glaciers as self-organized critical systems. Nat. Geosci. 7, 874–878. https://doi.org/10.1038/ngeo2290

Bittig, H.C., Maurer, T.L., Plant, J.N., Schmechtig, C., Wong, A.P.S., Claustre, H., Trull, T.W., Udaya Bhaskar, T.V.S., Boss, E., Dall'Olmo, G., Organelli, E., Poteau, A., Johnson, K.S., Hanstein, C., Leymarie, E., Le Reste, S., Riser, S.C., Rupan, A.R., Taillandier, V., Thierry, V., Xing, X., 2019. A BGC-Argo Guide: Planning, Deployment, Data Handling and Usage. Front. Mar. Sci. 6, 502. https://doi.org/10.3389/fmars.2019.00502

Del Castillo, C.E., Signorini, S.R., Karaköylü, E.M., Rivero-Calle, S., 2019. Is the Southern Ocean Getting Greener? Geophys. Res. Lett. 46, 6034–6040. https://doi.org/10.1029/2019GL083163

Dierssen, H.M., Smith, R.C., Vernet, M., 2002. Glacial meltwater dynamics in coastal waters west of the Antarctic peninsula. Proc. Natl. Acad. Sci. 99, 1790–1795. https://doi.org/10.1073/pnas.032206999

Fraser, A.D., Massom, R.A., Handcock, M.S., Reid, P., Ohshima, K.I., Raphael, M.N., Cartwright, J., Klekociuk, A.R., Wang, Z., Porter-Smith, R., 2021. Eighteen-year record of circum-Antarctic landfast-sea-ice distribution allows detailed baseline characterisation and reveals trends and variability. The Cryosphere 15, 5061–5077. https://doi.org/10.5194/tc-15-5061-2021

Fraser, A.D., Massom, R.A., Michael, K.J., 2010. Generation of high-resolution East Antarctic landfast sea-ice maps from cloud-free MODIS satellite composite imagery. Remote Sens. Environ. 114, 2888–2896. https://doi.org/10.1016/j.rse.2010.07.006

Fraser, A.D., Massom, R.A., Ohshima, K.I., Willmes, S., Kappes, P.J., Cartwright, J., Porter-Smith, R., 2020. High-resolution mapping of circum-Antarctic landfast sea ice distribution, 2000–2018. Earth Syst. Sci. Data 12, 2987–2999. https://doi.org/10.5194/essd-12-2987-2020

Fraser, A.D., Wongpan, P., Langhorne, P.J., Klekociuk, A.R., Kusahara, K., Lannuzel, D., Massom, R.A., Meiners, K.M., Swadling, K.M., Atwater, D.P., Brett, G.M., Corkill, M., Dalman, L.A., Fiddes, S., Granata, A., Guglielmo, L., Heil, P., Leonard, G.H., Mahoney, A.R., McMinn, A., van der Merwe, P., Weldrick, C.K., Wienecke, B., 2023. Antarctic Landfast Sea Ice: A Review of Its Physics, Biogeochemistry and Ecology. Rev. Geophys. 61, e2022RG000770. https://doi.org/10.1029/2022RG000770

Frenzel, H., Sharp, J.D., Fassbender, A.J., Buzby, N., 2022. OneArgo-Mat: A MATLAB toolbox for accessing and visualizing Argo data. https://doi.org/10.5281/zenodo.7055484

Google Trends, 2024. Antarctic sea ice.

Haëntjens, N., Boss, E., Talley, L.D., 2017. Revisiting Ocean Color algorithms for chlorophyll a and particulate organic carbon in the Southern Ocean using biogeochemical floats. J. Geophys. Res. Oceans 122, 6583–6593. https://doi.org/10.1002/2017JC012844

Hobday, A.J., Alexander, L.V., Perkins, S.E., Smale, D.A., Straub, S.C., Oliver, E.C.J., Benthuysen, J.A., Burrows, M.T., Donat, M.G., Feng, M., Holbrook, N.J., Moore, P.J., Scannell, H.A., Gupta, A.S., Wernberg, T., 2016. A hierarchical approach to defining marine heatwaves. Prog. Oceanogr. 141, 227–238. https://doi.org/10.1016/j.pocean.2015.12.014

Hu, C., Lee, Z., Franz, B., 2012. Chlorophyll *a* algorithms for oligotrophic oceans: A novel approach based on three-band reflectance difference. J. Geophys. Res. Oceans 117, 2011JC007395. https://doi.org/10.1029/2011JC007395

Johnson, R., Strutton, P.G., Wright, S.W., McMinn, A., Meiners, K.M., 2013. Three improved satellite chlorophyll algorithms for the Southern Ocean: SOUTHERN OCEAN CHLOROPHYLL ALGORITHMS. J. Geophys. Res. Oceans 118, 3694–3703. https://doi.org/10.1002/jgrc.20270

Kahru, M., Mitchell, B.G., 2010. Blending of ocean colour algorithms applied to the Southern Ocean. Remote Sens. Lett. 1, 119–124. https://doi.org/10.1080/01431160903547940

Kauko, H.M., Hattermann, T., Ryan-Keogh, T., Singh, A., De Steur, L., Fransson, A., Chierici, M., Falkenhaug, T., Hallfredsson, E.H., Bratbak, G., Tsagaraki, T., Berge, T., Zhou, Q., Moreau, S., 2021. Phenology and

Environmental Control of Phytoplankton Blooms in the Kong Håkon VII Hav in the Southern Ocean. Front. Mar. Sci. 8, 623856. https://doi.org/10.3389/fmars.2021.623856

Liniger, G., Strutton, P.G., Lannuzel, D., Moreau, S., 2020. Calving Event Led to Changes in Phytoplankton Bloom Phenology in the Mertz Polynya, Antarctica. J. Geophys. Res. Oceans 125, e2020JC016387. https://doi.org/10.1029/2020JC016387

Massonnet, F., Barreira, S., Barthélemy, A., Bilbao, R., Blanchard-Wrigglesworth, E., Blockley, E., Bromwich, D.H., Bushuk, M., Dong, X., Goessling, H.F., Hobbs, W., Iovino, D., Lee, W.-S., Li, C., Meier, W.N., Merryfield, W.J., Moreno-Chamarro, E., Morioka, Y., Li, X., Niraula, B., Petty, A., Sanna, A., Scilingo, M., Shu, Q., Sigmond, M., Sun, N., Tietsche, S., Wu, X., Yang, Q., Yuan, X., 2023. SIPN South: six years of coordinated seasonal Antarctic sea ice predictions. Front. Mar. Sci. 10, 1148899. https://doi.org/10.3389/fmars.2023.1148899

McCarthy, A.H., Peck, L.S., Aldridge, D.C., 2022. Ship traffic connects Antarctica's fragile coasts to worldwide ecosystems. Proc. Natl. Acad. Sci. 119, e2110303118. https://doi.org/10.1073/pnas.2110303118

Meier, W.N., Fetterer, F., Windnagel, A.K., Stewart, J.S., 2021a. NOAA/NSIDC Climate Data Record of Passive Microwave Sea Ice Concentration, Version 4. https://doi.org/10.7265/efmz-2t65

Meier, W.N., Fetterer, F., Windnagel, A.K., Stewart, J.S., 2021b. Near-Real-Time NOAA/NSIDC Climate Data Record of Passive Microwave Sea Ice Concentration, Version 2. https://doi.org/10.7265/tgam-yv28

Paul, S., Hendricks, S., Rinne, E., Sallila, H., 2021. Sea Ice Thickness Algorithm Theoretical Basis Document (ATBD), v2.0, ESA Climate Change Initiative on Sea Ice (SICCI).

Pereira, E.S., Garcia, C.A.E., 2018. Evaluation of satellite-derived MODIS chlorophyll algorithms in the northern Antarctic Peninsula. Deep Sea Res. Part II Top. Stud. Oceanogr. 149, 124–137. https://doi.org/10.1016/j.dsr2.2017.12.018

Reid, P.A., Massom, R.A., 2022. Change and variability in Antarctic coastal exposure, 1979–2020. Nat. Commun. 13, 1164. https://doi.org/10.1038/s41467-022-28676-z

Schmechtig, C., Claustre, H., Poteau, A., D'Ortenzio, F., Schallenberg, C., Trull, T., Xing, X., 2023. BGC-Argo quality control manual for the Chlorophyll-A concentration. Ifremer. https://doi.org/10.13155/35385

Sirjacobs, D., Alvera-Azcárate, A., Barth, A., Lacroix, G., Park, Y., Nechad, B., Ruddick, K., Beckers, J.-M., 2011. Cloud filling of ocean colour and sea surface temperature remote sensing products over the Southern North Sea by the Data Interpolating Empirical Orthogonal Functions methodology. J. Sea Res. 65, 114–130. https://doi.org/10.1016/j.seares.2010.08.002

Stammerjohn, S.E., Martinson, D.G., Smith, R.C., Yuan, X., Rind, D., 2008. Trends in Antarctic annual sea ice retreat and advance and their relation to El Niño–Southern Oscillation and Southern Annular Mode variability. J. Geophys. Res. 113, 1–20. https://doi.org/10.1029/2007JC004269

Thomalla, S.J., Nicholson, S.-A., Ryan-Keogh, T.J., Smith, M.E., 2023. Widespread changes in Southern Ocean phytoplankton blooms linked to climate drivers. Nat. Clim. Change 13, 975–984. https://doi.org/10.1038/s41558-023-01768-4

Wang, M., 2003. Light scattering from the spherical-shell atmosphere: Earth curvature effects measured by SeaWiFS. Eos Trans. Am. Geophys. Union 84, 529–534. https://doi.org/10.1029/2003EO480003

Xing, X., Briggs, N., Boss, E., Claustre, H., 2018. Improved correction for non-photochemical quenching of in situ chlorophyll fluorescence based on a synchronous irradiance profile. Opt. Express 26, 24734. https://doi.org/10.1364/OE.26.024734
